# A Spatial Multiomics Atlas and Resource for Immune-Fibrotic Niches in Chronic Kidney Allograft Dysfunction

**DOI:** 10.34133/cbsystems.0720

**Published:** 2026-11-05

**Authors:** Qiongqiong Gao, Zongpan Ke, Wenhao Xu, Ming Zheng, Bo Wang, Runmin Ding, Jiawen Liu, Zhou Hang, Ze Yu, Ke Ni, Huidong Su, Mulong Du, Mingyang Jiang, Dingwei Ye, Ruoyun Tan, Min Gu, Zijie Wang, Zeping Gui

**Affiliations:** ^1^ Department of Urology, The First Affiliated Hospital of USTC, Division of Life Sciences and Medicine, University of Science and Technology of China, Hefei 230002, China.; ^2^ Department of Urology, Jiangsu Key Laboratory of Urological Disease Prevention and Treatment, Institute for Organ Injury and Repair, The Second Affiliated Hospital of Nanjing Medical University, Nanjing Medical University, Nanjing 210003, China.; ^3^ The Second Clinical Medical College, Nanjing Medical University, Nanjing 211166, China.; ^4^ Department of Urology, Fudan University Shanghai Cancer Center, Shanghai 200032, China.; ^5^ Department of Urology, The First Affiliated Hospital of Nanjing Medical University, Nanjing Medical University, Nanjing 210036, China.; ^6^ RagOmics, RageneBio Inc., Verona, New Jersey 07044, USA.; ^7^ Department of Paediatrics and Adolescent Medicine, LKS Faculty of Medicine, The University of Hong Kong, Hong Kong SAR, China.; ^8^ Department of Biostatistics, Center for Global Health, School of Public Health, Nanjing Medical University, Nanjing 211166, Jiangsu, China.; ^9^ Department of Bone and Joint Surgery, The First Affiliated Hospital of Guangxi Medical University, Nanning 510120, China.

## Abstract

Chronic allograft dysfunction (CAD) is a major cause of late kidney-allograft failure, yet its spatially organized cellular and metabolic mechanisms remain poorly resolved because human biopsies are scarce and experimental models are imperfect. Here, we established a tacrolimus-modulated accelerated mouse model of CAD and constructed a cross-species multiomics atlas by integrating mouse single-cell RNA sequencing, flow cytometry, and multiplex imaging with human single-nucleus RNA sequencing, Visium HD spatial transcriptomics, and spatial metabolomics. Cross-species analysis revealed conserved immune, stromal, epithelial, and myeloid programs and delineated 2 anatomically distinct fibrotic niches. Cortical fibrosis was associated with endothelial/pericyte remodeling, altered cholesterol-biosynthesis transcriptional programs, and reduced regional cholesterol-related signals. Medullary fibrosis was enriched for proximal-tubule S3 [PT(S3)] cells with glycolysis-associated remodeling, increased regional lactate-related signals, and adjacent tissue-resident memory T-cell domains displaying an atypical exhaustion-like program. Glutamine/glutamate metabolism-associated programs accompanied inferred monocyte-to-macrophage state remodeling. In renal tubular epithelial cells, 2-deoxy-D-glucose partially attenuated transforming growth factor-β1-induced E-cadherin loss and α-smooth muscle actin induction, providing focused functional support for glycolysis-associated tubular remodeling. We additionally provide an exploratory kidney-transplant multiomics resource for data visualization, auxiliary summarization of platform-derived results, and gene/pathway querying. Together, this study provides a spatially resolved, cross-species framework for identifying candidate immune-metabolic mechanisms and prioritizing experimentally testable targets in CAD.

## Introduction

Kidney transplantation is the most effective treatment for end-stage renal disease and substantially extends patient survival [1]. Although short-term kidney-allograft survival is favorable, long-term outcomes remain limited by chronic allograft dysfunction (CAD) [2,3]. CAD encompasses persistent immune and nonimmune injury, inflammation, microvascular and tubular damage, and progressive extracellular matrix (ECM) accumulation that can culminate in graft failure [4,5]. Current immunosuppressive regimens reduce acute rejection but do not eliminate late graft injury and carry risks of infection, malignancy, and drug toxicity [5–7].

Renal interstitial fibrosis is a critical determinant of long-term outcome in CAD [8]. Its common pathological basis is abnormal ECM deposition in the renal interstitium, and myofibroblasts (MFBs) are major effector cells in this process [9]. However, the origins, phenotypic heterogeneity, and regulation of MFBs remain incompletely defined [10]. Although tubular epithelial cells and endothelial cells (ECs) have been proposed as potential contributors to the MFB pool in renal allograft fibrosis in our previous work, recent studies [11,12] indicate that MFB origin is context-dependent and more complex than previously appreciated. Depending on the pathological setting, MFBs may arise from multiple cellular compartments, including fibroblasts, pericytes, ECs, epithelial cells, and possibly macrophage-associated mesenchymal programs [10,13]. Recent lineage-tracing and human single-cell studies support fibroblast- and pericyte-lineage cells as the dominant established sources of matrix-producing MFBs in kidney fibrosis; we therefore interpret EMT (Epithelial-Mesenchymal Transition)-, EndMT (Endothelial-Mesenchymal Transition)-, and macrophage-associated mesenchymal programs more cautiously as context-dependent or partial transition states rather than major lineage contributions proven in vivo.

Animal models are essential for dissecting chronic alloimmune injury [14]. In our untreated C57BL/6-to-BALB/c cohort, recipients developed fulminant early rejection, preventing chronic-phase analysis. We therefore used a tacrolimus (Tac) induction-and-intermittent-maintenance schedule as an experimental compromise to suppress early lethal rejection while allowing later fibrotic remodeling. This regimen was not designed to reproduce continuous oral, trough-guided Tac exposure in clinical transplantation. The resulting system is referred to here as a Tac-modulated accelerated chronic allograft injury model. Conventional slow-rejection models can require many months to develop fibrosis, and injury kinetics vary substantially with strain direction and organ context [14–19].

Here, we combined a Tac-modulated CAD mouse model with single-cell profiling, flow cytometry, multiplex imaging, human single-nucleus RNA sequencing, and region-matched spatial transcriptomic and metabolomic analyses of adjacent biopsy sections to construct a cross-species spatial multiomics atlas of chronic allograft injury. This integrated framework enabled us to identify conserved cell states, define region-specific immune-fibrotic niches, and prioritize metabolic programs associated with disease progression. Cross-modal convergence across single-cell, spatial, imaging, and flow-cytometric analyses, together with focused perturbation of the glycolysis-associated tubular axis, strengthened the biological relevance of the identified mechanisms. We also provide an exploratory kidney-transplant multiomics resource for data visualization and gene/pathway exploration.

## Methods

Detailed experimental and computational procedures are provided in Supplementary Methods. The Tac-modulated Allo-CAD group was designed to suppress early lethal rejection and permit chronic-phase analysis; it was not intended to reproduce clinical Tac pharmacokinetics. Allo-SR (slow rejection) served as a descriptive comparator because its strain direction and immunosuppression differed from those of Allo-CAD. Lesion domains were scored using terminology adapted from Banff categories for semiquantitative murine comparison, not for assignment of a formal human Banff diagnosis. RNA velocity, pseudotime and partition-based graph abstraction (PAGA) were used to infer transcriptional state relationships; SpatialCellChat, CellChat, and CellPhoneDB were used to predict expression-based communication; and gene set variation analysis (GSVA), AUCell, and scMetabolism were used to calculate relative gene set or pathway scores. None of these outputs was treated as independent causal evidence.

Kidney-transplant multiomics resource. The platform hosts processed datasets, visualization modules, and reproducible workflow components. Automated functions provide structured summaries of platform-derived outputs and conversational gene/pathway querying for exploratory research. The supported data types, visual workflow, cross-species analysis modules, and auxiliary querying functions are summarized in Fig. S10.

In vitro glycolysis-inhibition experiment. HK-2 cells obtained from American Type Culture Collection were cultured in RPMI 1640 medium supplemented with fetal bovine serum (VivaCell) at 37 °C in 5% CO_2_. Cells were treated for 48 h with recombinant transforming growth factor-β1 (TGF-β1) (5 ng/ml; Sino Biological, 80116-RNAH), 2-DG (2-deoxy-D-glucose) (1 mM; MedChemExpress, HY-13966), both agents, or vehicle. E-cadherin and alpha-smooth muscle actin (α-SMA) were assessed by immunofluorescence. Fibronectin, lactate dehydrogenase A (LDHA), and α-SMA in renal grafts were assessed by immunoblotting with β-actin as the loading control, and SLC22A7, LDHA, and fibronectin were examined by multiplex tissue immunofluorescence. Proteintech antibodies were used at the manufacturer-recommended dilutions. Full procedures are provided in Supplementary Methods.

Human cohorts and spatial correspondence. The human analyses combined public and in-house datasets with source-specific metadata completeness. In-house spatial transcriptomics used Visium HD on formalin-fixed, paraffin-embedded biopsy sections, analyzed at 8- and 16-micrometer bins. MALDI-MSI was performed on adjacent 10-μm serial sections at 50-μm pixel resolution. Cross-modal comparisons were therefore performed at the level of matched anatomical regions rather than as same-section or single-cell coregistration [20,21]. Individual Banff lesion variables were not uniformly available across all public datasets.

## Results

### Characterization of a Tac-modulated accelerated CAD model

We compared 4 renal transplant settings (Fig. 1A). Syn, defined as C57BL/6-to-C57BL/6 transplantation, served as the syngeneic control. The Tac-modulated Allo-CAD group, defined as C57BL/6-to-BALB/c transplantation, received Tac daily for the first 7 d and weekly thereafter until day 105 to enable chronic-phase observation. Allo-SR, defined as untreated BALB/c-to-C57BL/6 transplantation, served as a slow-rejection benchmark. Allo-AR used the same C57BL/6-to-BALB/c strain direction as Allo-CAD but received no Tac and developed fulminant acute rejection in the present cohort. Because Allo-SR differed from Allo-CAD in both donor–recipient strain direction and immunosuppressive exposure, it was interpreted as a descriptive comparator of injury kinetics rather than a strain- and treatment-matched control.

**Fig. 1.**
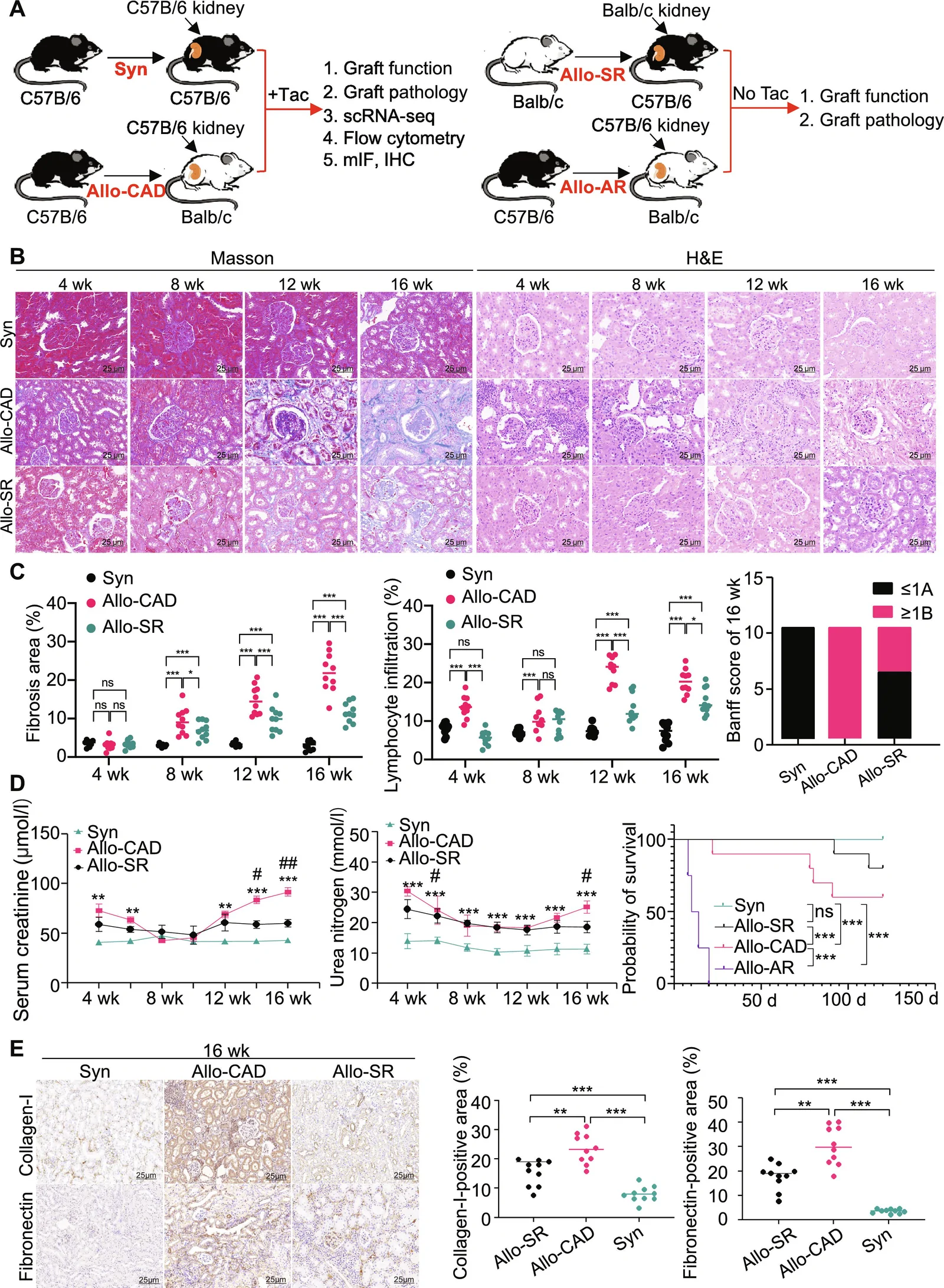
Characterization of a tacrolimus-modulated accelerated chronic allograft dysfunction model. (A) Experimental design showing donor–recipient strain combinations, tacrolimus treatment schedules, sampling time points, and downstream analyses. (B) Representative Masson’s trichrome and hematoxylin and eosin (H&E) staining of renal grafts at 4, 8, 12, and 16 weeks after transplantation. (C) Semiquantitative fibrosis area and lymphocyte infiltration at 4, 8, 12, and 16 weeks, together with the distribution of adapted murine rejection grades at 16 weeks. These adapted grades were used only for descriptive comparison in mice and do not constitute a formal human Banff diagnosis. (D) Longitudinal serum creatinine and blood urea nitrogen levels and Kaplan–Meier recipient survival. (E) Representative immunohistochemical images and quantification of collagen I and fibronectin deposition at 16 weeks. Data are presented as means ± standard error of the mean (SEM); each point represents 1 biological replicate. Sample sizes were *n* = 6, comparisons among groups were analyzed using 1-way analysis of variance (ANOVA). Survival curves were compared using the log-rank test. ns, not significant; *P < 0.05; **P < 0.01; ***P < 0.001; #*P* < 0.05; ##*P* < 0.01 (*Allo-SR group vs Syn group; #Allo-CAD vs Syn). Syn, syngeneic transplantation; Allo-CAD, the tacrolimus-modulated allogeneic group; Allo-SR, untreated allogeneic slow-rejection comparator; Allo-AR, untreated allogeneic acute-rejection group; Tac, tacrolimus; BUN, blood urea nitrogen. scRNA-seq, single-cell RNA sequencing; IHC, immunohistochemistry.

Serial histological assessment showed progressive graft injury in the Tac-modulated Allo-CAD group at 4, 8, 12, and 16 weeks (Fig. 1B). By week 12, the grafts displayed intimal arteritis, perivascular lymphocytic infiltration, and patchy interstitial fibrosis. We describe these findings as chronic active rejection-like injury rather than a formal human Banff diagnosis. By week 16, the grafts showed widespread interstitial fibrosis, tubular atrophy, and focal global glomerulosclerosis, consistent with advanced chronic allograft injury. Allo-SR grafts showed slower pathological progression at the sampled time points. Fibrosis area and lymphocyte infiltration increased over time and were generally greater in Allo-CAD than in Syn and Allo-SR at the later time points (Fig. 1C). Lesion domains adapted from Banff terminology were used only for descriptive comparison in mice and were not used to assign a formal clinical Banff category. Serum creatinine and blood urea nitrogen measurements indicated earlier graft dysfunction in Allo-CAD, and collagen I and fibronectin deposition was greater in Allo-CAD at 16 weeks than in the comparator groups (Fig. 1D and E).

Most untreated C57BL/6-to-BALB/c recipients in the Allo-AR cohort died within 2 weeks. At the acute comparison time point, Tac-modulated Allo-CAD grafts showed lower interstitial inflammation and tubulitis-like lesion scores and reduced CD4^+^ and CD8^+^ T-cell infiltration compared with Allo-AR grafts (Fig. S1). Together with recipient survival beyond the acute phase and the subsequent development of fibrosis and tubular atrophy, these findings indicate that the later Allo-CAD phenotype cannot be explained solely by persistence of untreated fulminant acute rejection. Nevertheless, the model represents 1 donor–recipient strain direction and 1 immunosuppressive schedule. Its chronic active rejection-like features should therefore not be generalized to all forms of chronic kidney allograft injury.

### Cross-species identification of conserved cell states and pathways in chronic allograft injury

We assembled mouse single-cell RNA sequencing and human single-cell or single-nucleus RNA sequencing datasets from in-house experiments and public Gene Expression Omnibus and European Molecular Biology Laboratory-European Bioinformatics Institute resources, encompassing CAD, antibody-mediated rejection (ABMR), and stable kidney allografts [22,23]. Quality-control metrics of the integrated human and mouse datasets, including sequencing quality, detected features, and retained cell distributions, are summarized in Fig. S2A to C. SATURN was used to integrate the datasets in a shared macrogene space, enabling cross-species comparison beyond strict one-to-one ortholog matching [24]. Because macrogene-based integration can accentuate shared programs and attenuate species-specific differences, the integrated embedding was used to assess broad correspondence rather than quantitative equivalence. Subsequent endothelial, epithelial, T-cell, and myeloid analyses were performed within species and then compared across modalities.

Major human and mouse cell classes showed broad correspondence in the shared embedding while retaining species-specific structure (Fig. 2B). Condition-level distributions and relationships among stable, CAD, ABMR, Syn, and Allo-CAD samples are shown in Fig. S2D to F. At the level of major cell classes, mouse Allo-CAD grafts showed broad correspondence with human CAD samples. ABMR samples remained more distinct, whereas Syn grafts were closer to stable transplant samples than to normal kidney in the condition-level comparison (Fig. S2D). Shared macrogene programs captured antigen-processing and immune-related functions in mononuclear phagocyte system (MPS) populations, together with translational, metabolic, cytoskeletal, and regulatory programs in T cells (Fig. 2C). Ingenuity Pathway Analysis enabled directional comparison of selected pathways across species and revealed both concordant and species-specific patterns in T-cell and MPS compartments (Fig. 2D). Together, these analyses identify partially conserved cell-state and pathway programs across human and mouse chronic allograft injury datasets while highlighting species- and cohort-specific differences.

**Fig. 2.**
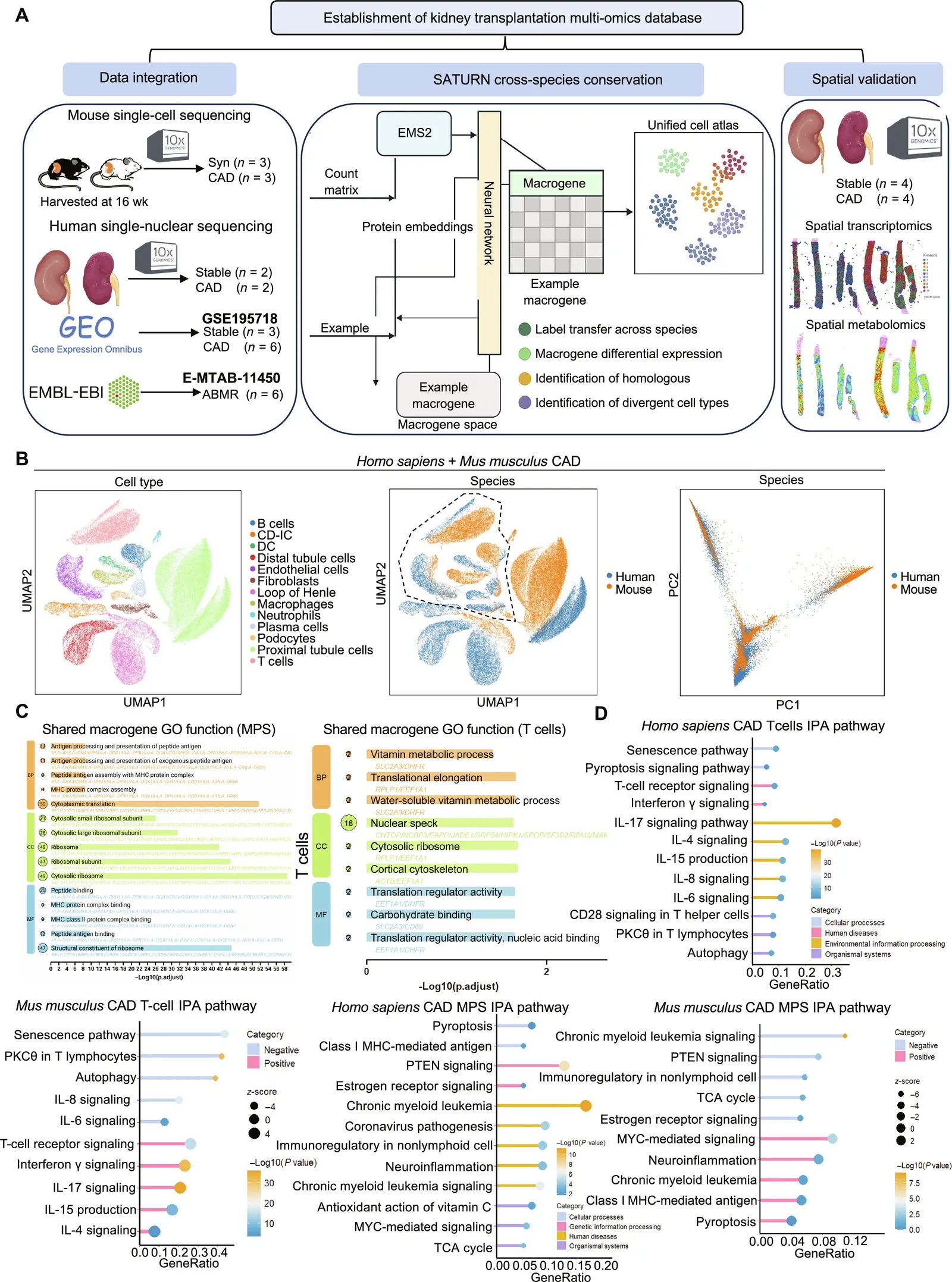
Study design and cross-species identification of conserved cell states and pathways. (A) Workflow integrating mouse single-cell RNA sequencing, human single-cell or single-nucleus RNA sequencing, SATURN-based cross-species analysis, and human spatial transcriptomic and metabolomic profiling within a kidney-transplant multiomics resource. (B) Integrated uniform manifold approximation and projection (UMAP) and principal component analysis views showing broad cell-class organization and the distributions of human and mouse cells. (C) Gene Ontology enrichment of shared macrogene programs in mononuclear phagocyte system (MPS) populations and T cells. (D) Directional comparison of selected Ingenuity Pathway Analysis (IPA) pathways in human and mouse T-cell and mononuclear phagocyte system compartments. ABMR, antibody-mediated rejection; GEO, Gene Expression Omnibus; GO, Gene Ontology; CD-IC, collecting duct-intercalated cell; DC, distal convoluted tubule.

### Spatial and single-cell mapping identifies region-specific candidate cellular contributors to ECM production

For subsequent mouse single-cell analyses, the Tac-modulated Allo-CAD group is abbreviated as Allo. We profiled renal grafts from 3 Syn and 3 Allo recipients at 16 weeks and retained 64,889 cells after quality control, including 36,079 Syn cells and 28,810 Allo cells (Fig. S3). The resulting atlas comprised epithelial, stromal, B-cell, plasma-cell, T-cell, NK-cell, neutrophil, and MPS compartments (Fig. 3A). Graph-based subclustering identified 17 transcriptional populations, including an unclassified population (Fig. 3B). Epithelial cells constituted the largest compartment in Syn grafts, whereas Allo grafts showed increased representation of immune populations (Fig. 3B and C). GSVA showed relative enrichment of interferon-γ response, interleukin 6/Janus kinase/signal transducer and activator of transcription 3 signaling and tumor necrosis factor-α signaling via nuclear factor-κB in several Allo immune compartments (Fig. 3E). These scores represent relative transcriptional enrichment within annotated populations and are not functional perturbation readouts.

**Fig. 3.**
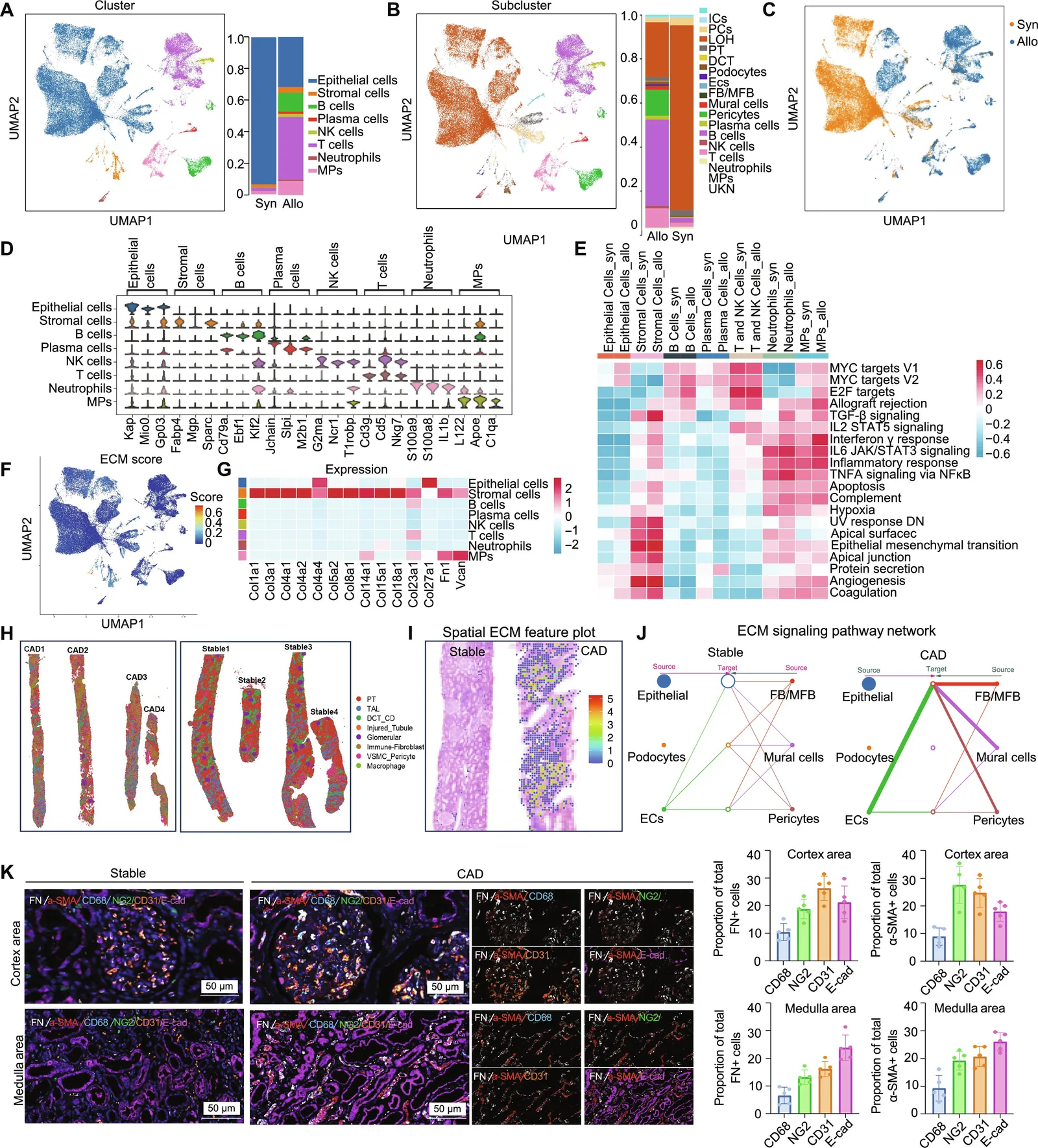
Spatial and single-cell mapping identifies region-specific candidate cellular contributors to extracellular matrix (ECM) production. (A) Uniform manifold approximation and projection (UMAP) visualization and relative proportions of 8 major cellular compartments in renal grafts from Syn and Allo recipients. (B) UMAP visualization and relative proportions of 17 transcriptional subclusters. (C) UMAP showing the distributions of Syn and Allo cells in the integrated mouse single-cell atlas. (D) Expression of representative marker genes across the major cellular compartments. (E) Relative gene set variation analysis scores for selected Hallmark gene sets across major cellular compartments in Syn and Allo grafts. (F) UMAP visualization of ECM-related AUCell scores across the mouse single-cell atlas. (G) Expression of selected ECM-associated genes across major cellular compartments. (H) Visium HD maps from stable and chronic allograft dysfunction (CAD) biopsies annotated according to dominant tubular, glomerular, immune-fibrotic, vascular/pericyte, and macrophage-associated spatial features. (I) Representative spatial feature maps showing regional ECM gene-set expression in stable and CAD biopsies. (J) CellChat-predicted ECM-related signaling networks among cell-type-enriched spatial domains in stable and CAD biopsies. (K) Representative multiplex immunofluorescence images showing fibronectin and α-SMA together with CD68, NG2, CD31, and E-cadherin in cortical and medullary regions of stable and CAD biopsies. Scale bars, 50 μm. The accompanying plots show the percentages of fibronectin (FN)-positive and α-SMA-positive cells colocalizing with the indicated cell-type markers in CAD. Each point represents 1 biopsy. Data are presented as means ± standard error of the mean (SEM). DCT, distal convoluted tubule; ECs, endothelial cells; FB/MFB, fibroblasts and myofibroblasts; ICs, intercalated cells; LOH, loop of Henle; MPS, mononuclear phagocyte system; NK, natural killer; PCs, principal cells; PT, proximal tubule; TAL, thick ascending limb; UKN, unclassified cells; VSMC, vascular smooth muscle cell.

An ECM-related gene set previously applied to human CAD was scored using AUCell. ECM-associated transcriptional activity was detected across multiple compartments, with the strongest signal in stromal populations and lower but detectable signals in selected epithelial and myeloid populations (Fig. 3F and G). These signals identify transcriptional states associated with fibrotic remodeling but do not establish lineage conversion or quantify deposited matrix. Human spatial ECM–gene signals represent coordinated transcription across multicellular domains and were interpreted alongside histological and protein-level evidence of matrix deposition.

Human spatial transcriptomic profiling included 4 stable and 4 CAD biopsies. Spatial bins were grouped according to dominant tissue and cell-state features, including proximal tubular, thick ascending limb, distal tubular, injured tubular, glomerular, immune-fibrotic, vascular smooth muscle/pericyte, and macrophage-enriched domains (Fig. 3H). The mixed immune-fibrotic domain was retained as a composite spatial category rather than being assigned a discrete single-cell identity. Representative spatial maps showed regionally increased ECM–gene expression in CAD biopsies (Fig. 3I). CellChat analysis predicted altered ECM-related communication among endothelial, pericyte, mural, epithelial, podocyte, and fibroblast (FB)/MFB-enriched spatial domains (Fig. 3J). These expression-based predictions do not validate specific ligand–receptor interactions. Multiplex immunofluorescence showed region-dependent colocalization patterns of fibronectin and α-SMA with endothelial, pericyte, epithelial, and macrophage markers (Fig. 3K). These spatial associations do not establish direct cell–cell contact, functional ligand–receptor signaling or lineage conversion.

### Endothelial and perivascular remodeling is associated with cholesterol-related program changes and cortical fibrosis

We jointly analyzed FB/MFB, ECs, pericytes, mural cells, and podocytes to characterize vascular and matrix-associated cell states. The relative representation of ECs was lower, whereas FB/MFB and pericytes were more highly represented in Allo grafts (Fig. 4A). GSVA showed condition-associated changes in inflammatory, TGF-β, mesenchymal-transition, and lipid-related gene-set scores across podocyte, endothelial, FB/MFB, and mural-cell populations (Fig. 4B). Pseudotime and RNA-velocity analyses suggested transcriptional relationships involving EC, pericyte, and FB/MFB states, accompanied by changes in collagen-associated and lipid-metabolic genes along pseudotime (Fig. 4C and D). These inferred trajectories do not demonstrate that ECs or pericytes convert into MFBs in vivo. Functional enrichment and regulon analyses further revealed subtype-specific transcriptional programs across vascular and matrix-associated populations, including ECs, FB/MFB, pericytes, podocytes, and mural cells (Fig. S4).

**Fig. 4.**
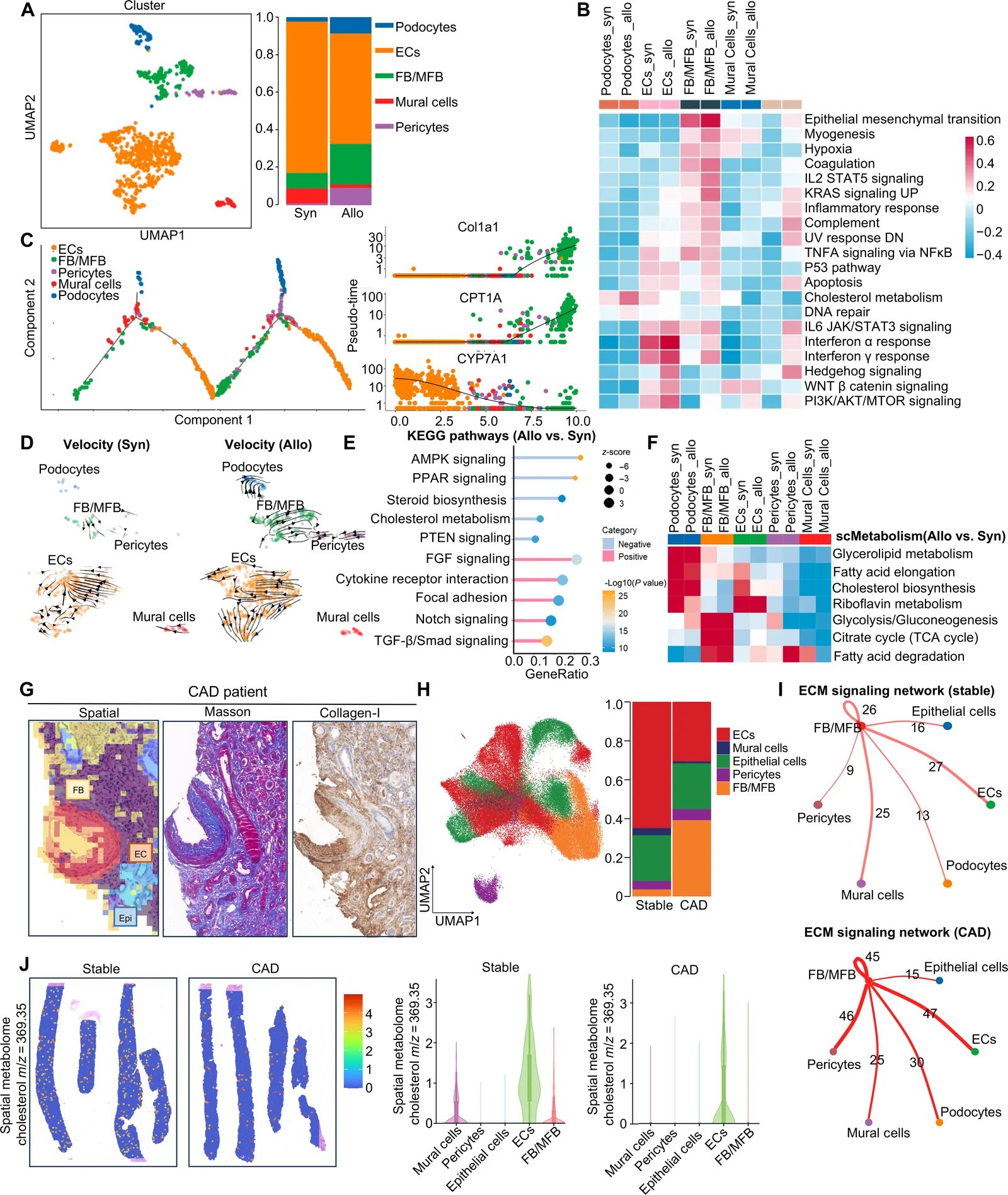
Endothelial and perivascular remodeling is associated with cholesterol-related program changes and cortical fibrosis. (A) Uniform manifold approximation and projection (UMAP) visualization and relative proportions of fibroblasts and myofibroblasts (FB/MFB), endothelial cells, pericytes, mural cells, and podocytes in Syn and Allo grafts. (B) Relative gene set variation analysis scores for selected Hallmark gene sets in podocyte, endothelial, FB/MFB, and mural-cell populations from Syn and Allo grafts. (C) Pseudotime trajectory of the analyzed vascular and matrix-associated populations, with pseudotime-dependent expression patterns of Col1a1, Cpt1a, and Cyp7a1. (D) RNA-velocity embeddings showing inferred transcriptional state relationships in Syn and Allo grafts. The velocity analyses do not establish lineage conversion in vivo. (E) Kyoto Encyclopedia of Genes and Genomes (KEGG) enrichment of pseudotime-associated genes. (F) Relative scMetabolism pathway scores across the indicated populations in Syn and Allo grafts. These scores are expression based and do not directly measure metabolic flux. (G) Representative chronic allograft dysfunction (CAD) cortical region showing spatially annotated domains and corresponding Masson’s trichrome and collagen I staining on matched tissue sections. (H) UMAP visualization and relative composition of cell-type-enriched bins in selected cortical spatial domains from stable and CAD biopsies. (I) CellChat-predicted FB/MFB-centered extracellular matrix (ECM)-related signaling networks in stable and CAD cortical domains. (J) The spatial metabolomics maps of a cholesterol-related ion signal at mass/charge ratio (*m*/*z*) 369.35 in stable and CAD cortical regions, with signal distributions across matched spatial domains enriched for the indicated cell types.

Kyoto Encyclopedia of Genes and Genomes (KEGG) enrichment and scMetabolism analyses identified relative differences in AMPK, steroid- and cholesterol-related, glycolytic, and fatty acid metabolic programs across the analyzed populations (Fig. 4E and F). These results represent expression-based pathway enrichment and do not directly measure metabolic flux or cholesterol synthesis. Consistent with lineage-tracing studies, fibroblast- and pericyte-lineage populations remain the best-established sources of renal MFBs.

Representative human CAD cortical regions showed spatial adjacency between FB/MFB-enriched and EC-enriched domains (Fig. 4G). Across the selected cortical spatial domains, CAD samples contained a higher relative proportion of FB/MFB-enriched bins and a lower proportion of EC-enriched bins than stable samples (Fig. 4H). CellChat predicted stronger FB/MFB-centered ECM-related communication with EC-enriched and pericyte-enriched domains in CAD (Fig. 4I). These communication networks are expression-based predictions and do not validate specific ligand–receptor interactions.

Analysis by spatial metabolomics on adjacent serial sections showed lower intensity of a cholesterol-related ion signal in CAD cortical regions (Fig. 4J). Because Visium HD and spatial metabolomics were acquired on adjacent sections and at different spatial resolutions, the metabolite signals assigned to cell-type-enriched domains were interpreted as correspondence between matched anatomical regions rather than cell-intrinsic measurements. Together, these findings support an association among endothelial and perivascular remodeling, lower regional cholesterol-related signals, and cortical fibrosis.

### PT(S3)-enriched glycolysis-associated transcriptional remodeling is associated with medullary fibrosis in CAD

Epithelial subclustering identified intercalated cells, principal cells, loop of Henle cells, distal convoluted tubular cells, and 2 proximal-tubule populations. PT1 expressed S1-S2 markers, whereas PT2 expressed S3-associated markers; we therefore designated PT1 as PT(S1-2) and PT2 as PT(S3) (Fig. 5A and Fig. S5). Across epithelial populations, GSVA showed condition-associated differences in injury and inflammatory gene-set scores, including TGF-β and tumor necrosis factor-α/nuclear factor-κB signaling (Fig. 5B). AUCell and ECM–gene expression analyses showed increased ECM-associated transcriptional scores in Allo PT(S3) cells (Fig. 5C and D). Joint RNA-velocity and pseudotime analyses placed PT(S3) and FB/MFB states within connected branches of inferred transcriptional manifolds (Fig. 5E and F). These analyses identify a fibrosis-associated epithelial stress state but do not demonstrate epithelial conversion into MFBs in vivo.

**Fig. 5.**
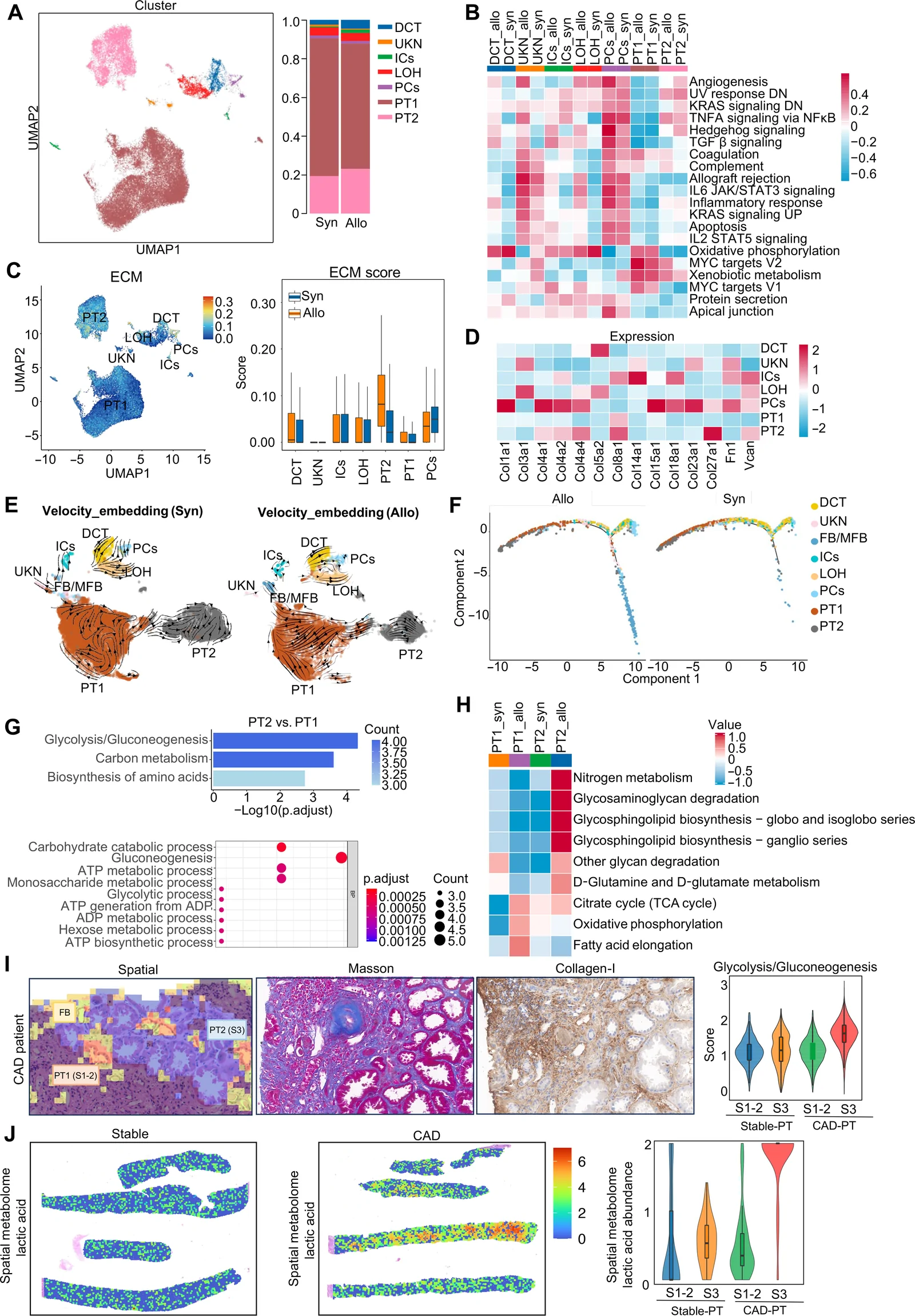
PT(S3)-enriched glycolysis-associated programs characterize medullary fibrotic niches in chronic allograft dysfunction (CAD). (A) Uniform manifold approximation and projection (UMAP) visualization and relative proportions of epithelial populations in Syn and Allo grafts, including distal convoluted tubular cells (DCTs), intercalated cells (ICs), loop of Henle cells (LOH), principal cells (PCs), PT(S1-2), PT(S3), and an unclassified population. (B) Relative gene set variation analysis scores for selected Hallmark gene sets across epithelial populations in Syn and Allo grafts. (C) UMAP visualization of extracellular matrix (ECM)-related AUCell scores and summary scores across epithelial populations in Syn and Allo grafts. (D) Relative expression of selected ECM-associated genes across epithelial populations in Allo and Syn grafts. (E) RNA-velocity embeddings showing inferred local transcriptional dynamics among epithelial and fibroblast and myofibroblast (FB/MFB) states in Syn and Allo grafts. (F) Pseudotime trajectories showing inferred transcriptional state relationships among epithelial and FB/MFB populations. (G) Kyoto Encyclopedia of Genes and Genomes (KEGG) and Gene Ontology enrichment analyses comparing PT(S3) with PT(S1-2). (H) Relative scMetabolism pathway scores across PT(S1-2) and PT(S3) populations from Syn and Allo grafts. These scores were expression-based and did not directly measure metabolic flux. (I) Representative human CAD medullary Visium HD map showing PT1(S1-2)-enriched, PT2(S3)-enriched, and FB/MFB-enriched domains, with corresponding Masson’s trichrome and collagen I staining and glycolysis/gluconeogenesis score distributions in stable and CAD spatial bins. (J) The spatial metabolomics maps showing a lactate-related ion signal in representative stable and CAD medullary sections, together with signal distributions across anatomically matched PT(S1-2)-enriched and PT(S3)-enriched regions. DCT, distal convoluted tubule; PT, proximal tubule; TCA, tricarboxylic acid cycle; UKN, unclassified cells.

Compared with PT(S1-2), PT(S3) was enriched for glycolysis/gluconeogenesis, carbon metabolism, carbohydrate catabolism, and adenosine triphosphate-metabolic processes (Fig. 5G). scMetabolism further showed condition-associated differences in tricarboxylic acid-cycle, oxidative-phosphorylation, glutamine/glutamate, glycan, and fatty acid-related transcriptional scores across PT subsets (Fig. 5H). Medullary Visium HD data showed PT(S3)-enriched bins adjacent to FB/MFB-enriched ECM-rich domains, with elevated glycolysis/gluconeogenesis score distributions in CAD PT(S3)-enriched bins (Fig. 5I). Analysis by spatial metabolomics performed on adjacent serial sections showed higher regional lactate-related ion intensity in CAD medullary regions (Fig. 5J). Because the assays were performed on adjacent sections at different spatial resolutions, these findings were interpreted as regional concordance between PT(S3)-enriched transcriptional domains and lactate-related signals rather than as direct measurement of lactate within individual PT(S3) cells.

To provide focused perturbation-based support for the glycolysis-associated tubular axis, HK-2 cells were treated with TGF-β1 at 5 ng/ml for 48 h. The groups received vehicle, TGF-β1 (5 ng/ml), 2-DG (1 mM), or TGF-β1 plus 2-DG. TGF-β1 reduced E-cadherin and increased α-SMA, whereas 2-DG partially attenuated both responses (Fig. S6A and B). Multiplex immunofluorescence showed stronger regional LDHA and fibronectin signals in SLC22A7-enriched tubular areas of Allo-CAD grafts, while whole-graft immunoblotting showed increased LDHA, fibronectin, and α-SMA relative to Syn grafts (Fig. S6C and D). Together, these findings provide functional evidence supporting glycolysis-associated tubular fibrotic remodeling but do not establish PT(S3)-restricted lineage conversion or in vivo metabolic causality [25,26].

Together, these findings identify PT(S3) as a tubular state associated with glycolysis-related transcriptional remodeling and medullary fibrotic niches. Cross-modal spatial profiling and glycolysis-inhibition experiments provide further support for this association but do not establish PT(S3)-restricted lineage conversion or in vivo metabolic causality.

### Trms display an exhaustion-like transcriptional program and localize near PT(S3)-enriched domains in CAD

T-cell subcluster distributions differed between Syn and Allo grafts, with greater relative representation of exhausted-like T-cell and tissue-resident memory T-cell populations in Allo grafts (Fig. 6A). GSVA revealed heterogeneous inflammatory, stress-response, cell-cycle, and junction-associated gene-set patterns across T-cell populations (Fig. 6B). CellPhoneDB predicted a larger number of interaction pairs between CD8Trm and epithelial populations in Allo than in Syn grafts (Fig. 6C), with additional predicted immune-interaction patterns shown in Fig. S7A.

**Fig. 6.**
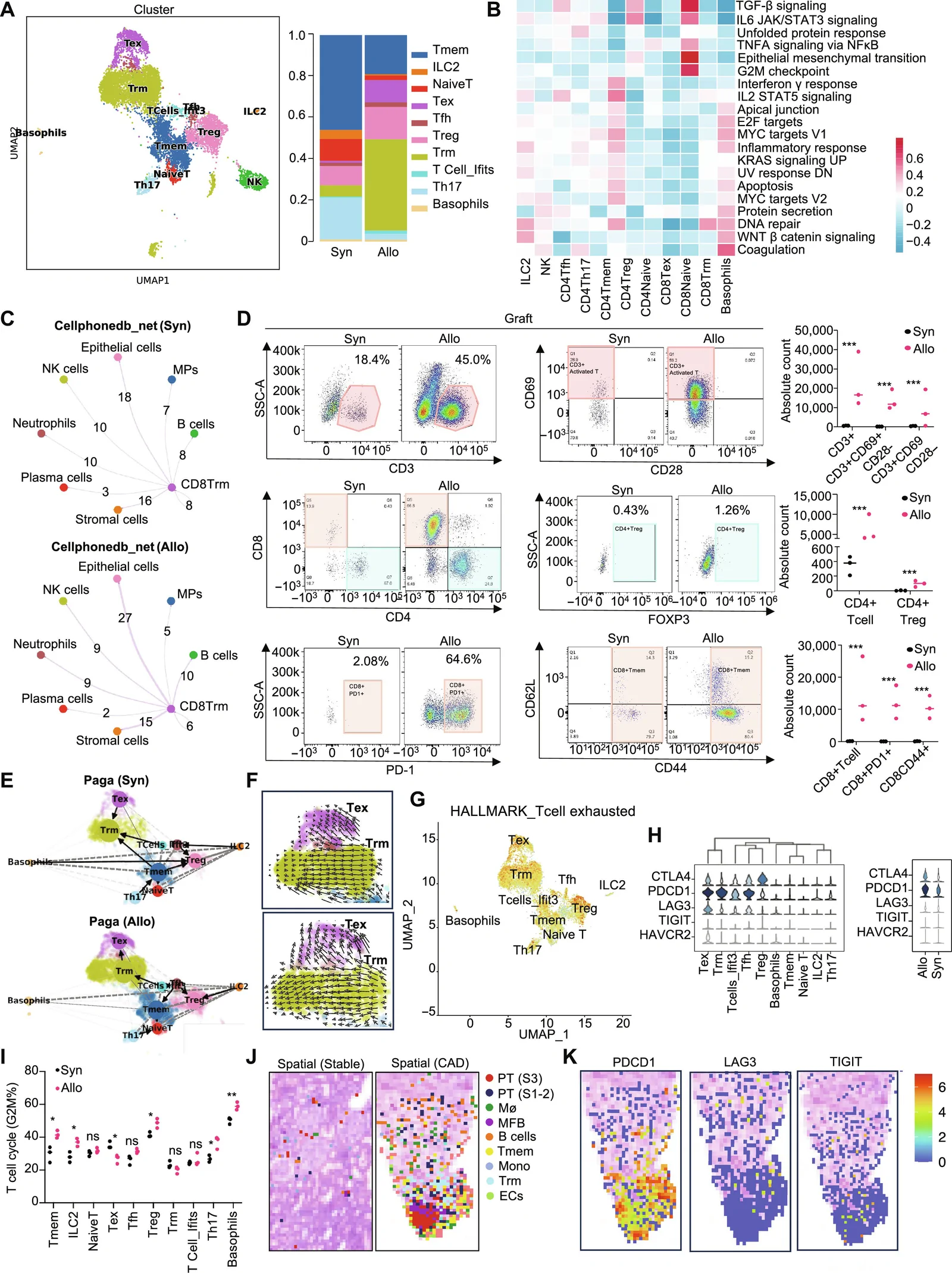
Tissue-resident memory T cells display an exhaustion-like transcriptional program and localize near PT(S3)-enriched domains in chronic allograft dysfunction (CAD). (A) Uniform manifold approximation and projection (UMAP) visualization and relative proportions of T-cell subpopulations in Syn and Allo grafts. (B) Relative gene set variation analysis scores for selected gene sets across T-cell populations. (C) CellPhoneDB-predicted interaction networks between CD8Trm and major cellular compartments in Syn and Allo grafts. (D) Representative flow cytometry plots and absolute counts of graft-infiltrating T-cell populations expressing the indicated activation and checkpoint-associated markers. Each point represents 1 biological replicate. (E) Partition-based graph abstraction (PAGA) graphs showing inferred connectivity among T-cell transcriptional states in Syn and Allo grafts. (F) RNA-velocity embeddings showing inferred local transcriptional dynamics of tissue-resident memory T cell (Trm) and exhausted-like T cell (Tex). (G) UMAP visualization of the T-cell exhaustion-associated transcriptional score. (H) Expression of selected checkpoint-associated genes across T-cell populations and between Allo and Syn grafts. (I) G2M-phase representation across T-cell populations in Syn and Allo grafts. (J) Representative spatial maps showing PT(S3)-enriched, PT(S1-2)-enriched, myofibroblast (MFB)-enriched, Trm-enriched, and other cell-type-enriched bins in stable and CAD biopsies. (K) Regional expression of PDCD1, LAG3, and TIGIT in the representative CAD spatial domain. ns, not significant,*P < 0.05; **P < 0.01; ***P < 0.001. ILC2, group 2 innate lymphoid cell; Tfh, follicular helper T cell; Th17, T helper 17 cell; Tmem, memory T cell; Treg, regulatory T cell.

Flow cytometry provided additional evidence of graft-localized T-cell remodeling, including increased absolute counts of T-cell populations bearing activation or checkpoint-associated markers and increased CD4^+^ regulatory T cell counts in Allo grafts (Fig. 6D). The specific checkpoint marker is reported as programmed cell death protein 1 after verification against the antibody panel and original flow cytometry files. The spleen showed less pronounced differences (Fig. S7B).

PAGA and RNA-velocity analyses suggested a shift in the inferred transcriptional relationship between tissue-resident memory T cell (Trm) and exhausted-like T cell (Tex)-like populations, with trajectories predominantly oriented from Tex-like toward Trm in Syn grafts but from Trm toward Tex-like states in Allo grafts (Fig. 6E and F). An exhaustion-associated transcriptional score and checkpoint-gene expression were enriched in Tex-like and Trm populations, including expression of programmed cell death 1, cytotoxic T-lymphocyte-associated protein 4, and lymphocyte activation gene 3 (Fig. 6G and H). Additional checkpoint-related gene-expression patterns across T-cell populations are shown in Fig. S7C. We therefore describe these populations as displaying an exhaustion-like transcriptional program rather than functional exhaustion. The inferred state relationships do not establish a unidirectional transition from Trm to Tex-like cells. Cell-cycle analysis showed subtype-specific and condition-associated differences in G2M-phase representation but did not provide a functional proliferation measurement (Fig. 6I).

In representative human CAD spatial domains, PT(S3)-enriched, MFB-enriched, and Trm-enriched bins occurred in regional proximity, with localized programmed cell death 1 expression in the same broader domain (Fig. 6J and K).

Together, these findings identify a Trm-associated immune niche characterized by an exhaustion-like transcriptional program, predicted epithelial communication, and regional proximity to PT(S3)-enriched fibrotic domains. While these observations support coordinated immune remodeling in chronic allograft injury, they do not establish direct Trm-mediated epithelial regulation, functional exhaustion, or profibrotic causality.

### Myeloid-state remodeling is associated with glutamine/glutamate-related transcriptional programs in CAD

MPS subclustering identified macrophages, monocytes, monocyte-derived dendritic cells, conventional dendritic cells, and plasmacytoid dendritic cells (Fig. 7A). Conventional dendritic cells were further separated into cDC1 and cDC2 states for pathway analysis. The relative representation of macrophages was lower, whereas monocytes and conventional dendritic cells were more highly represented in Allo grafts. GSVA showed enrichment of inflammatory, complement, TGF-β, and epithelial–mesenchymal-transition-associated programs in macrophage and monocyte compartments, whereas E2F, G2M-checkpoint, and MYC-related programs were more prominent in conventional dendritic-cell subsets (Fig. 7B). G2M-phase assignments showed modest condition-associated increases in macrophage and monocyte populations, with limited changes in dendritic-cell subsets (Fig. 7C). These patterns support substantial myeloid-state heterogeneity rather than a single uniform profibrotic macrophage program.

**Fig. 7.**
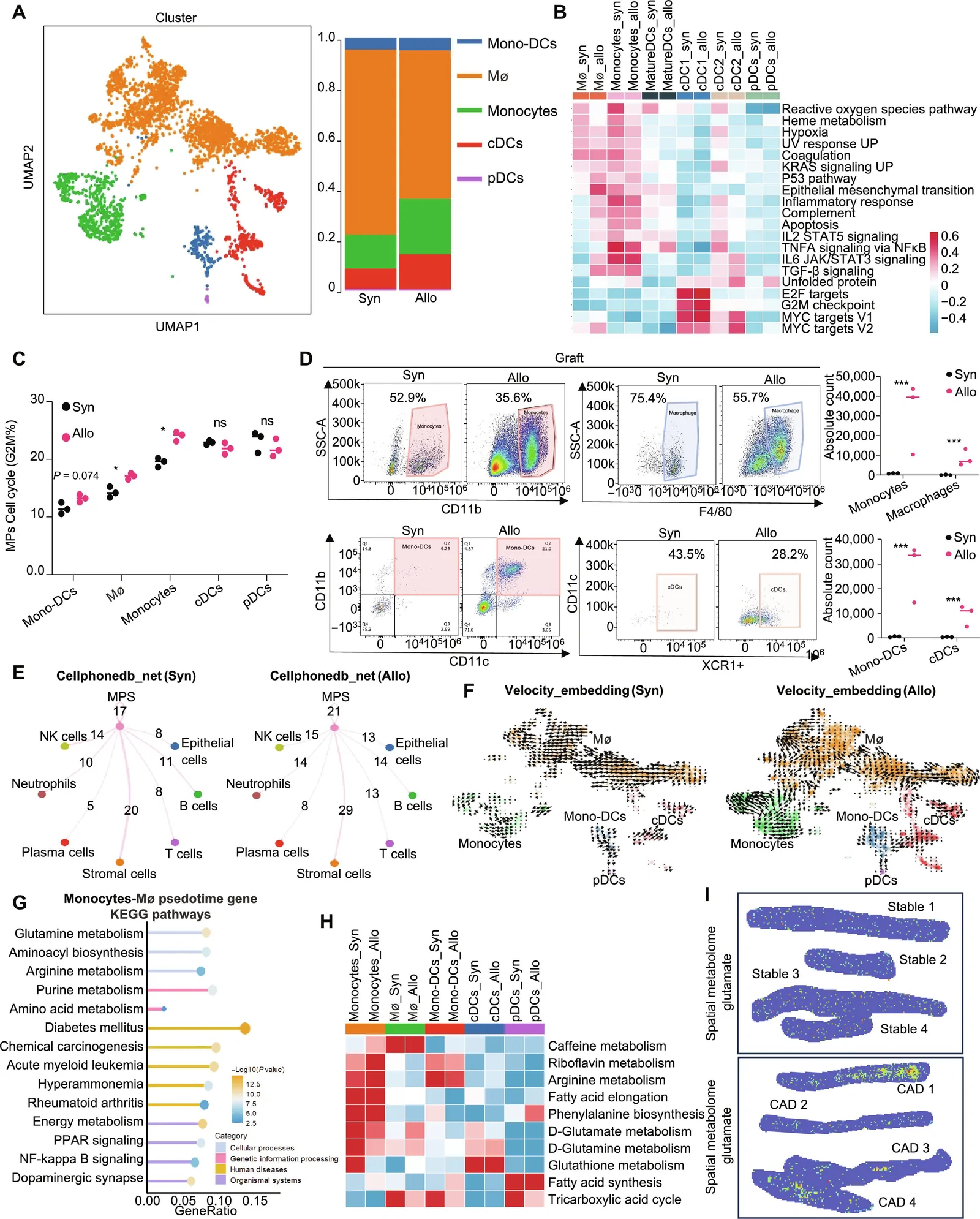
Myeloid-state remodeling is associated with glutamine/glutamate-related transcriptional programs in chronic allograft dysfunction (CAD). (A) Uniform manifold approximation and projection (UMAP) visualization and relative proportions of macrophages, monocytes, monocyte-derived dendritic cells, conventional dendritic cells (cDC), and plasmacytoid dendritic cells (pDCs) in Syn and Allo grafts. (B) Relative gene set variation analysis scores for selected gene sets across myeloid populations in Syn and Allo grafts. cDCs were separated into cDC1 and cDC2 states for pathway analysis. (C) G2M-phase representation across myeloid populations in Syn and Allo grafts. (D) Representative flow cytometry plots and absolute counts of graft-infiltrating monocytes, macrophages, monocyte-derived dendritic cells (Mono-DCs), and the indicated cDC population. Each point represents 1 biological replicate. (E) CellPhoneDB-predicted interaction networks involving mononuclear phagocyte system (MPS) and other major cellular compartments in Syn and Allo grafts. (F) RNA-velocity embeddings showing inferred local transcriptional dynamics among monocyte, macrophage, and dendritic-cell states. (G) Kyoto Encyclopedia of Genes and Genomes (KEGG) enrichment of genes associated with the inferred monocyte–macrophage pseudotime continuum. (H) Relative scMetabolism pathway scores across myeloid populations in Syn and Allo grafts. These scores were expression-based and do not directly measure metabolic flux. (I) Spatial metabolomic maps showing a glutamate-related signal in stable and CAD biopsy sections.

Flow cytometry showed increased absolute numbers of graft-infiltrating monocytes, macrophages, monocyte-derived dendritic cells, and the indicated conventional dendritic-cell population in Allo grafts, despite lower gated frequencies for several populations (Fig. 7D). These measurements provide additional evidence for graft-localized myeloid remodeling.

CellPhoneDB predicted a larger number of interaction pairs involving MPS and several epithelial and immune compartments in Allo than in Syn grafts (Fig. 7E). RNA-velocity analysis placed monocyte, macrophage, and dendritic-cell states within connected transcriptional neighborhoods, with condition-associated differences in local velocity patterns (Fig. 7F). These expression-based and trajectory-based analyses do not establish functional cell–cell communication or monocyte-to-macrophage differentiation in vivo.

KEGG enrichment of pseudotime-associated genes implicated glutamine and broader amino-acid metabolic programs along the inferred monocyte–macrophage continuum (Fig. 7G). scMetabolism further showed subtype- and condition-associated differences in D-glutamine, D-glutamate, glutathione, arginine, and tricarboxylic acid-cycle-related transcriptional scores (Fig. 7H). Spatial metabolomic maps of human biopsy sections showed more prominent regional glutamate-related ion signals in several CAD sections than in stable sections (Fig. 7I).

To further resolve monocyte heterogeneity, we examined Ly6c1, a transcript associated with Ly6C-high inflammatory monocyte states. Ly6c1 was broadly detected across MPS populations but was most highly expressed in monocytes (Fig. S8A and B). Monocytes were therefore subdivided into Ly6c1-high and Ly6c1-low states, with a greater relative representation of the Ly6c1-high state in Allo grafts (Fig. S7C). Monocle2, RNA velocity, and PAGA placed the 2 states along an inferred transcriptional continuum in Allo grafts (Fig. S8D to F). pySCENIC analysis further identified condition-associated differences in regulon activity related to immune activation and cell-state heterogeneity in T-cell and MPS compartments (Fig. S9).

Together, these findings identify heterogeneous myeloid-state remodeling accompanied by glutamine/glutamate-related transcriptional changes and regional glutamate-related signals in CAD. These observations identify a potential link between immune remodeling and metabolism but do not establish metabolic control of myeloid differentiation or profibrotic function.

## Discussion

This study integrates a Tac-modulated mouse allograft model with mouse single-cell profiling and human single-nucleus, spatial transcriptomic and spatial metabolomic datasets. The atlas identifies spatially organized immune, stromal, epithelial and myeloid programs associated with chronic allograft injury. Its principal contributions are a map integrating multiple modalities and a set of testable hypotheses. The atlas does not establish causality for each inferred transition or pathway.

The Allo-CAD group permitted observation beyond the fulminant acute phase and developed progressive fibrosis, tubular atrophy, and functional impairment within 16 weeks. It should be interpreted as a Tac-modulated accelerated chronic allograft injury model established in a defined C57BL/6-to-BALB/c strain direction and immunosuppressive schedule, rather than as a direct experimental equivalent of clinical CAD. Allo-SR served only as a descriptive comparator of injury kinetics because it differed from Allo-CAD in both donor–recipient strain direction and Tac exposure. Accordingly, the comparison between these groups was not used to isolate Tac-specific or strain-specific effects. Murine lesion scores were adapted from Banff terminology for semiquantitative pathological comparison and were not used to assign a formal human Banff diagnosis. In the present cohort, most untreated C57BL/6-to-BALB/c recipients died within 2 weeks, whereas Allo-CAD recipients survived beyond the acute phase. At the acute comparison time point, Allo-CAD grafts showed lower interstitial inflammation, lower tubulitis-like lesion scores, and reduced CD4+ and CD8+ T-cell infiltration relative to untreated Allo-AR grafts. The subsequent emergence of fibrosis and tubular atrophy indicates that the later Allo-CAD phenotype cannot be explained solely by persistence of untreated fulminant acute rejection. However, these findings do not exclude ongoing chronic active alloimmune injury. Because rejection kinetics depend on strain direction, immunosuppressive exposure, surgical conditions, and experimental context, the untreated C57BL/6-to-BALB/c outcome observed here should not be regarded as a universal property of this strain combination [16–18,27].

Cross-species SATURN integration identified broad conserved programs, while key endothelial, epithelial, T-cell, and myeloid observations were derived within species and evaluated across modalities [28]. This strategy reduces reliance on the integrated representation but does not eliminate cohort heterogeneity or species-specific effects. The human datasets were limited and incompletely annotated for lesion-level Banff variables, disease duration, donor type, mixed rejection, infection and calcineurin-inhibitor toxicity.

The myeloid analyses associate monocyte/macrophage state remodeling with transcriptional scores associated with glutamine/glutamate metabolism, and the T-cell analyses identify a graft-localized Trm-enriched exhaustion-like program. These associations are biologically plausible in light of prior work on inflammatory metabolism, macrophage remodeling, and Trm states during persistent antigen exposure [24,29], but direct perturbation is required to determine necessity, sufficiency, and functional directionality [30,31].

Two regional patterns emerged. Cortical fibrotic regions showed endothelial/pericyte-associated remodeling and lower regional cholesterol-related signals, whereas medullary fibrotic regions showed PT(S3)-enriched glycolysis-associated programs and higher regional lactate-related signals. Spatial transcriptomics and MALDI-MSI were acquired on adjacent serial sections at different spatial scales, so these findings represent region-level correspondence rather than same-cell metabolic measurements [20,21]. Fibroblast- and pericyte-lineage cells remain the best-established sources of renal MFBs; endothelial and epithelial findings are interpreted as transition-associated cell states within fibrotic niches. Focused 2-DG treatment in HK-2 cells partially reversed TGF-β1-induced E-cadherin loss and α-SMA induction, providing perturbation-based support for the glycolysis-associated tubular axis. This direction is consistent with prior studies linking TGF-β1, tubular glycolysis, and fibrotic remodeling [25,26], but it does not establish PT(S3)-specific or in vivo causality.

The study has several limitations. First, the model uses 1 strain direction and 1 Tac schedule and does not isolate strain, Tac, and chronic-injury effects. Second, most trajectory, communication, and pathway analyses remain observational. The 2-DG experiment supports a role for glycolysis in tubular remodeling, but no lineage tracing, adoptive transfer, in vivo pathway-specific perturbation, or functional validation of the other nominated axes was performed. Third, the human cohorts are small and incompletely harmonized. Fourth, spatial correspondence is based on adjacent sections and region-level matching. Fifth, the kidney-transplant multiomics resource is intended for data exploration, workflow execution, automated summarization, and conversational gene/pathway querying. Its automated functions were not benchmarked as predictive models and were not developed for clinical prediction, diagnosis or treatment recommendations. Future work should prioritize lineage-resolved perturbation, expanded Banff-annotated cohorts, longer follow-up, and spatially coregistered assays. The in vitro experiment addresses some, but not all, of the causal uncertainty in the atlas findings.

In summary, the atlas identifies conserved and regionally organized cell-state and metabolic programs associated with chronic allograft injury. The glycolysis-inhibition experiment provides functional evidence supporting a role for glycolysis in tubular remodeling, while the broader atlas remains a framework for testing endothelial/pericyte, PT(S3), Trm, and myeloid hypotheses.

## Ethical Approval

The studies involving human participants were approved by the Ethics Committee of the Second Affiliated Hospital of Nanjing Medical University (approval number: 2023-SRFA-007). All participants provided written informed consent. All animal experiments were conducted according to protocols approved by the Animal Research Ethics Committee of Nanjing Medical University (approval number: IACUC-2109025).

## Data Availability

Raw and processed sequencing data generated in this study are deposited in the Gene Expression Omnibus under accession GSE300341. Platform documentation is available at https://ragomics-doc.readthedocs.io/en/latest/. The interactive resource is an exploratory research interface and does not currently have a DOI or versioned release. Additional analysis materials can be obtained from the corresponding authors upon reasonable request, subject to institutional and ethical requirements.
