# Supplementary material for: A Spatial Multiomics Atlas and Resource for Immune-Fibrotic Niches in Chronic Kidney Allograft Dysfunction
Source: Cyborg Bionic Syst. 2026 Nov 5;7:0720. doi: 10.34133/cbsystems.0720 (PMC13735209; doi:10.34133/cbsystems.0720)
Supplement: Supplementary file 1 [file cbsystems.0720.f1.zip › 260913-Supplemental Figure 1-10.docx]

**Supplemental Figures and Legends**

**Figure S1. Histological comparison of untreated acute rejection and Tac-modulated Allo-CAD injury. (A)** Representative histological images (H&E and Masson’s trichrome staining) showing severe lymphocyte infiltration, extensive fibrosis, and structural damage in the acute rejection (Allo-AR) model compared with the Tac-treated Allo-CAD model at 4 weeks post-transplantation. **(B-C)** Quantification of Banff scores for lymphocyte infiltration, interstitial inflammation, and tubulitis in renal allografts from Allo-AR and Allo-CAD models at 4 weeks post-transplantation. **(D-E)** Representative IHC staining and quantification of CD8⁺ and CD4⁺ T-cell infiltration in renal allografts from Allo-AR, Allo-CAD, and Syn groups at 4 weeks post-transplantation. **(F)** Representative whole-kidney histological images stained with HE and Masson's trichrome among Syn and Allo-CAD models at 12 weeks post-transplantation. Data were presented as mean ± SEM. ns, no significant; *, *p* < 0.05; **, *p* < 0.01; ***, *p* < 0.001. Statistical analysis in panels C-E was performed by one-way ANOVA with multiple comparisons.

**Figure S2. Quality control and cell type distribution in cross-species single-cell RNA-seq datasets. (A-C)** Quality control steps for integrated human and mouse scRNA-seq datasets, including removal of low-quality cells and sequencing artifacts. **(D-F)** Comparison of cell type proportions across normal stable, CAD, and ABMR groups in both human and CAD mouse model datasets following integration, highlighting species-specific distribution patterns.

**Figure S3. Quality control metrics for scRNA-seq samples**. **(A-F)** Violin plot displaying the number of detected genes per cell (nFeature_RNA) across the dataset. Violin plot showing the total RNA counts per cell (nCount_RNA) across the dataset. Violin plot representing the percentage of mitochondrial gene expression per cell (Percent.mt) across the dataset. These metrics provided an overview of the cellular RNA quality and mitochondrial gene contribution for the 6 scRNA-seq samples.

**Figure S4. Functional enrichment and regulon patterns across vascular and matrix-associated populations between Syn and Allo grafts. (A)** Bar plot displaying GO Biological Process enrichment analysis results of different stromal cell subtypes. **(B)** Bubble plot displaying KEGG enrichment analysis results of different stromal cell subtypes. **(C)** pySCENIC analysis revealing transcription factor regulon specificity scores in different stromal cell subtypes between the Syn and Allo groups. **(D)** Heatmap of regulon specificity scores for transcription factors in different stromal cell subtypes between the Syn and Allo groups.

**Figure S5. Feature plots of S3-associated marker genes in the PT(S3) epithelial population.**

**Figure S6. Glycolytic inhibition attenuates tubular EMT-like remodeling in Allo-CAD. (A)** Experimental validation strategy. **(B)** Representative immunofluorescence images and quantification of E-cadherin and α-SMA in HK-2 cells treated with vehicle, recombinant human TGF-β1 , 2-deoxy-D-glucose (2-DG), or TGF-β1 plus 2-DG. 2-DG partially attenuated TGF-β1-induced E-cadherin loss and α-SMA upregulation. **(C)** Multiplex immunofluorescence of Syn and Allo-CAD grafts showing Fibronectin, LDHA, SLC22A7 and DAPI. **(D)** Western blot and quantification of Fibronectin, LDHA and α-SMA, normalized to β-actin. Data were mean ± SEM; n = 3 independent experiments or biological samples. Scale bars, 50 μm.

**Figure S7. Comprehensive analysis of T cell interactions, exhaustion markers, and spleen populations. (A)** Heatmap showing cell-cell interaction ligands and receptors among T-cell subsets from CellPhoneDB analysis. **(B)** Flow cytometry analyses of spleen-derived T cells, comparing the activation states and Treg proportions between the Syn and Allo groups. **(C)** UMAP feature plots showing expression of the checkpoint-associated genes CTLA4, PDCD1, LAG3, TIGIT, and HAVCR2 across T-cell subpopulations in the mouse single-cell dataset. Data were presented as mean ± SEM. Ns, no significant. Statistical analysis in panel C was performed by one-way ANOVA with multiple comparisons.

**Figure S8. Analysis of Ly6C1-associated monocyte states and inferred transcriptional relationships. (A-C)** UMAP visualization, expression, and proportion plots of Ly6C1-high and Ly6C1-low monocyte subsets comparing Syn and Allo conditions. **(D-F)** Monocle2, RNA velocity, and PAGA analyses demonstrating differentiation trajectories and dynamics of Ly6C1-high monocytes under alloimmune stimulation. Data were presented as mean ± SEM. Ns, no significant. Statistical analysis in panel C was performed by one-way ANOVA with multiple comparisons.

**Figure S9. The results of transcription factor enrichment analysis on T cells and MPs. (A)** pySCENIC analysis revealing transcription factor regulon specificity scores in different MP subtypes. **(B)** pySCENIC analysis revealing transcription factor regulon specificity scores in different T cell subtypes. **(C)** Heatmap of transcription factor activity in different MPs subtypes highlighting key biological pathways. **(D)** Heatmap of transcription factor activity in different T cell subtypes highlighting key biological pathways.

**Figure S10.** **Overview of the kidney-transplant multi-omics resource. (A)** Overview of supported data formats, omics modalities, analytical toolkits, and modular function blocks. The resource supports commonly used single-cell and spatial omics data formats, including FASTQ, 10x Cell Ranger outputs, AnnData, and RDS files, and integrates scRNA-seq/snRNA-seq, scATAC-seq, and spatial omics datasets. Built-in analysis modules include preprocessing, quality control, visualization, batch correction, clustering, cell annotation, trajectory inference, RNA dynamics analysis, and functional enrichment analysis. **(B)** Visual workflow interface for modular analysis construction. Public and imported datasets can be organized within the platform, and analysis pipelines can be assembled by connecting predefined function blocks. The workflow structure enables reproducible and traceable execution of multi-step analyses without requiring direct programming. The embedded assistant provides auxiliary functions for structured output summarization and gene/pathway querying to facilitate exploratory data interpretation.
