# Supplementary material for: A Spatial Multiomics Atlas and Resource for Immune-Fibrotic Niches in Chronic Kidney Allograft Dysfunction
Source: Cyborg Bionic Syst. 2026 Nov 5;7:0720. doi: 10.34133/cbsystems.0720 (PMC13735209; doi:10.34133/cbsystems.0720)
Supplement: Supplementary file 1 [file cbsystems.0720.f1.zip › Supplementary_Methods_and_Materials_20260913.docx]

**Methods**

**Kidney Transplant Model and Experimental Procedures**

All animal experiments were conducted with approval from the Animal Research Ethics Committee of Nanjing Medical University (approval number: IACUC-2109025). Renal allogeneic transplantation models were established as previously described (1). In particular, renal transplantation was performed under inhaled isoflurane anesthesia (2% in O2) using aseptic technique. Donor kidneys were perfused with cold saline and the left kidney (with a short aortic cuff) was harvested from the donor. In the recipient, end-to-side microsurgical anastomoses were created between the donor aortic cuff and the recipient abdominal aorta, and between the donor renal vein and the recipient inferior vena cava, using 10-0 nylon sutures under an operating microscope. The donor ureter was implanted into the recipient’s bladder (ureteroneocystostomy) to re-establish urinary continuity. Post-operative care included subcutaneous buprenorphine for analgesia and daily monitoring of body weight, hydration, and activity. Graft viability was confirmed by adequate urine output and stable or improving postoperative weight, and rejection was monitored by serial serum creatinine measurements.

Mice were anesthetized using isoflurane inhalation (RWD Life Science). Subsequently, the donor mouse's left kidney was transplanted into the recipient. The average total ischemia time during the procedure ranged from 40 to 60 minutes.

**Syn and Tac-Modulated Allo-CAD Mouse Models**

For the syngeneic control (Syn), C57BL/6 kidneys were transplanted into C57BL/6 recipients. For the Tac-modulated Allo-CAD group, C57BL/6 kidneys were transplanted into BALB/c recipients. Contralateral nephrectomy was performed seven days after transplantation. Tacrolimus (Astellas Pharma) was administered intraperitoneally at 1.5 mg/kg daily for the first seven days and then weekly through week 16. Grafts were collected at 4, 8, 12 and 16 weeks for histologic, molecular and single-cell analyses. This regimen was designed to suppress early lethal rejection and permit chronic-phase observation; it was not intended to reproduce clinical trough-guided Tac exposure.

**Allo-SR model**

To model classical slow rejection, BALB/c mice served as allogeneic donors, and C57BL/6 mice were used as recipients without tacrolimus treatment. Contralateral nephrectomies were performed seven days post-transplantation. Chronic rejection features typically developed over a prolonged observation period, requiring more than 16 weeks for fibrosis and rejection to manifest. Kidneys were collected for histological and molecular analysis at defined time points.

**Allo-AR model**

For the untreated acute-rejection comparator (Allo-AR), C57BL/6 kidneys were transplanted into BALB/c recipients without Tac. In the present cohort, all untreated recipients died within two weeks. Grafts were collected 10-14 days after transplantation to characterize the fulminant acute phase.

**Histopathology, Immunohistochemistry, and Adapted Murine Lesion Scoring**

Renal grafts collected at 4, 8, 12, and 16 weeks were fixed in 10% neutral-buffered formalin, paraffin embedded, and sectioned at 4 μm. Sections were stained with hematoxylin and eosin and Masson’s trichrome for evaluation of inflammatory injury and interstitial fibrosis. Fibrotic area and lymphocyte infiltration were quantified from five nonoverlapping fields per graft using ImageJ and expressed as percentages of the evaluable tissue area.

For immunohistochemistry, sections underwent antigen retrieval and were incubated with primary antibodies against collagen I and fibronectin, followed by horseradish peroxidase-conjugated secondary antibodies and 3,3′-diaminobenzidine detection. Positive staining was quantified using a fixed ImageJ threshold and expressed as the percentage of positively stained tissue area.

Murine graft injury was evaluated using semiquantitative lesion domains adapted from Banff terminology, including interstitial inflammation, tubulitis-like injury, vascular inflammation, interstitial fibrosis, and tubular atrophy. These adapted scores were used only for descriptive comparison between mouse groups and were not used to assign a formal human Banff diagnosis. The categories shown in Figure 1C summarize lower-grade and higher-grade adapted murine lesion severity at 16 weeks. Each animal was treated as one biological replicate, and histological assessments were performed without knowledge of the experimental group.

**Kidney-Transplant Multi-Omics Resource**

To support data reuse and reproducible single-cell and spatial analyses in kidney transplantation, we curated public and in-house CAD, ABMR and stable-graft datasets on the Ragomics platform. Raw and processed data types included mouse scRNA-seq, human snRNA-seq, Visium HD spatial transcriptomics and MALDI-MSI spatial metabolomics.

Datasets were converted to Ragomics-compatible objects, including count matrices, Seurat/AnnData objects and spatial coordinates, and were annotated with sample, condition, batch and modality metadata. The visual workflow interface supports quality control, clustering, annotation, trajectory analysis, communication analysis and pathway enrichment. Optional automated functions generate structured summaries of platform-derived outputs and support conversational gene/pathway queries; no predictive model was trained or benchmarked, and these functions are not intended for clinical prediction, diagnosis or treatment recommendation. A user tutorial is available at https://ragomics-doc.readthedocs.io/en/latest/. Raw sequencing data generated in this study are deposited in GEO under GSE300341. Processed objects and analysis scripts are available from the corresponding authors upon reasonable request and subject to applicable ethics and consent restrictions.

**Tissue digestion and flow cytometry strategy**

Mice were sacrificed, and kidney grafts and spleens were harvested for flow cytometry analysis. Single-cell suspensions were prepared from these tissues. For kidney grafts, tissues were minced into small pieces and digested using a mouse tumor dissociation kit (Miltenyi Biotec, 130-096-730). The tissue was incubated with enzyme mix (2.35 mL DMEM, 100 µL enzyme D, 50 µL enzyme R, and 12.5 µL enzyme A) in a C Tube and processed using a gentleMACS Dissociator. Digested tissues were filtered through a 70 µm cell strainer and centrifuged at 300 g for 10 minutes. Red blood cells and debris were removed, and single-cell suspensions were prepared. Spleens were homogenized by grinding through a 70 µm cell strainer using cold PBS. The cell suspension was centrifuged at 500 g for 5 minutes, and red blood cells were lysed using 1X BD Pharm Lyse™ lysing buffer (BD Biosciences, 555899). Cells were washed twice with PBS and resuspended for staining. For surface staining, cells were washed with PBS and resuspended at 1x10^6 cells/mL. Fc receptors were blocked with Fc Block (BD Biosciences, 553142) for 10 minutes at 4 °C. Cells were stained with fluorescently labeled antibodies for 30 minutes at 4°C. After surface staining, cells were fixed and permeabilized using BD Cytofix/Cytoperm™ kit (BD Biosciences, 554714) according to the manufacturer’s instructions. Cells were then stained with antibodies against intracellular markers for 30 minutes at 4°C. Samples were acquired on a BD LSRFortessa™ flow cytometer and analyzed using FlowJo software (Tree Star, Inc.). Compensation was performed using single-stained controls, and gating was based on fluorescence minus one (FMO) controls.

Flow cytometry was used to characterize graft and splenic immune populations. After exclusion of debris, doublets and nonviable cells, CD45-positive leukocytes were analyzed for T-cell and myeloid subsets. T cells were identified with CD3 and separated into CD4- and CD8-positive populations; CD69, CD28, PD-1, CD44, CD62L and Foxp3 were used to characterize activation, memory, checkpoint-positive and regulatory states. Monocytes, macrophages, monocyte-derived dendritic cells and conventional dendritic cells were identified using CD11b, F4/80, CD11c and XCR1. α-SMA staining in macrophage-gated cells was treated as mesenchymal-marker expression and was not interpreted as proof of macrophage-to-myofibroblast lineage conversion.

Spleen samples were analyzed using the same core gating strategy; representative results are shown in Figure S6C.

**Sample processing and single-cell isolation**

Renal grafts were diced into small pieces with a scalpel and then incubated at 37°C for 5 minutes in a freshly made dissociation buffer containing 2 mg/mL Collagenase P and 0.2 mg/mL DNase I. The dissociated tissue was collected and filtered through a 40 μm cell strainer into ice-cold PBS. Cells were then pelleted by centrifugation at 300 g for 5 minutes at 4°C. After resuspension in Red Blood Cell lysis buffer for 5 minutes, the cells were centrifuged again at 200 g for 5 minutes at 4°C and resuspended in PBS with 0.04% BSA (ThermoFisher Scientific). The suspension was passed through a 40-μm cell strainer to eliminate cell clumps and large fragments. Cell count and viability were assessed using the Luna Cell counter, following previously established protocols. Libraries for single-cell RNA sequencing (scRNA-seq) were prepared using the Chromium Single Cell 5′ Library and Gel Bead & Multiplex Kit (10x Genomics), targeting approximately 5,000 cells per library if dissociation yielded sufficient cell numbers. All libraries were sequenced on an Illumina NextSeq until achieving adequate saturation. Post-quality control, raw sequencing reads were aligned to the mouse reference genome GRCm38 and processed into a matrix of unique molecular identifiers (UMIs) per cell barcode per gene using CellRanger (10x Genomics, v3.1).

**Spatial Transcriptomics (10x Visium HD)**

Formalin-fixed paraffin-embedded (FFPE) biopsy samples from kidney transplant patients underwent initial histological examination and quality control (QC) for RNA integrity, ensuring DV200 values exceeded 30%. Qualified samples were subjected to spatial transcriptomics using the 10x Genomics Visium HD platform, following the manufacturer's protocol. Tissue sections (5-10 μm thickness) were prepared on specialized Visium slides containing approximately 11 million capture probes, each spatially barcoded with unique molecular identifiers (UMIs). Sections underwent probe hybridization and subsequent library construction. Sequencing was performed using the Illumina NovaSeq system in paired-end 150 bp mode, targeting approximately 50,000 to 100,000 reads per 16 μm bin. Raw sequence data were processed with SpaceRanger software (version 3.0) utilizing STAR alignment to map sequences against the reference genome, producing spatial gene expression matrices at resolutions of 8 μm and 16 μm. Data normalization, dimensionality reduction, clustering analysis, and differential gene expression analysis were conducted using Seurat (version 5.1.0). Pathway and functional annotations were performed using clusterProfiler, referencing GO, KEGG, and Reactome databases. Spatial distribution and clustering results were visualized through Uniform Manifold Approximation and Projection (UMAP) and heatmaps.

**Deep spatial metabolomics (timsTOF fleX MALDI-2 Mass Spectrometry Imaging)**

Adjacent tissue sections were analyzed for spatial metabolomics using the Bruker timsTOF fleX MALDI-2 mass spectrometry imaging (MSI) system. Tissues were sectioned at 10 μm thickness, mounted on conductive slides, and coated with NEDC matrix solution (70% methanol and 3.4% ammonia) using a TM-Sprayer (TMSP-M3). Mass spectra were acquired in negative-ion mode within a mass-to-charge (m/z) range of 50-1300 at a spatial resolution of 50 μm per pixel. Data acquisition involved a laser frequency of 10,000 Hz, energy at 70%, and accumulation of 200 laser shots per pixel. Acquired spectra were processed using SCiLS Lab software (version 2024b Pro), incorporating baseline correction, peak alignment, smoothing, and normalization (Root Mean Square normalization method). Metabolite identification was performed using Tidymass software with reference to HMDB databases, achieving mass accuracy within ±10 ppm. Spatially resolved metabolite data underwent dimensionality reduction (PCA), clustering (Graph-based Louvain algorithm), and UMAP visualization using Seurat (version 5.1.0). Differential metabolites were statistically assessed by Wilcoxon rank-sum tests with a significance threshold set at adjusted p-value ≤ 0.05 and log2 fold change ≥ 0.25.

**Quality control and single-cell data analysis**

FastQ files generated by 10x Genomics standard sequencing protocols were aligned to the mouse pre-mRNA reference sequence (build GRCm38) using CellRanger (10x Genomics, v3.1). Outputs were subjected to preliminary QC to evaluate detected features, gene expression depth, and mitochondrial gene expression proportion per sample. Post-QC, the integrated dataset was assessed for batch effects using the R package ‘Seurat’. Principal Component Analysis (PCA) followed by Uniform Manifold Approximation and Projection (UMAP) was used for cell clustering. Distinct cell clusters were identified based on the gene expression (GE) of highly variable genes. GE patterns were analyzed between clusters to identify cluster-specific gene markers, which were compared against a publicly available database of cell type-specific genes using gene set enrichment analysis. Major cell types and unidentified cell types with non-specific markers were determined, and cell type proportions were evaluated across different conditions. Subsequent analyses involved subclustering functionally relevant cell types and assessing differentially expressed genes (DEGs) for each cell type and their subclusters between different kidney graft conditions (FDR ≤ 0.05). All statistical analyses and visualizations were conducted using R.

Filtered gene expression matrices from each sample were merged and analyzed using Seurat V4. Cells were filtered out if they had fewer than 400 or more than 10,000 detected genes or if they had over 25% mitochondrial transcripts. After filtering, all objects were integrated using 3000 features. UMAP for the full dataset was generated using Seurat’s DimPlot function with the top 17 principal components. Clusters were created using the FindNeighbors and FindClusters functions in Seurat (resolution= 2). Cluster identification was performed using the FeaturePlot function by evaluating the expression of specific markers in each cluster. Dot plots, violin plots, and heatmaps were generated using Seurat’s DotPlot and VlnPlot functions, with normalized counts in the RNA assay as input. Enriched genes were identified using Seurat's FindMarkers function, with a p-value cut-off of < 0.05 to select significantly enriched genes in specific clusters. The expression levels of significantly enriched genes were calculated using Seurat's AverageExpression function, categorized by mouse group. A validation scRNA-seq dataset was compiled and analyzed as previously described.

**Integrated Analysis of Cell-Cell Communication Using CellChat and CellPhoneDB**

CellChat employs a mass action-based approach to quantify the probability of communication between ligands and their respective receptors, factoring in the cell group proportions and the expressed co-factors among all sequenced cells (https://github.com/sqjin/CellChat)(2). A Seurat object comprising all cells was converted into a CellChat object. The recently identified LILRA and LILRB ligand-receptor pairs were incorporated into the CellChat database. The overall cell-cell communication network was determined by counting the number of interactions or summarizing the communication probabilities. This network, representing the interaction strengths (weights) between all clusters, was illustrated using circle plots. Each ligand-receptor pair's contribution to the signaling pathways was calculated, and the function extractEnrichedLR was employed to identify significant interactions (L-R pairs) and associated signaling genes across available pathways. Arcs represent the communication pathways between cell populations, with the width and color of the lines indicating the strength and frequency of the interactions. The outer segments represent distinct cell types involved in the communication network. Communication scores were interpreted as expression-based predictions rather than experimentally validated ligand-receptor activity.

In parallel, CellPhoneDB (https://github.com/Teichlab/cellphonedb)(3) was utilized to deduce cell-cell communication networks from the same single-cell transcriptomic dataset. The Seurat object was formatted for compatibility with CellPhoneDB's input requirements, and CellPhoneDB's statistical method was applied to identify significant ligand-receptor interactions by generating a null distribution via random permutations of cell labels. The significance of the observed interactions was assessed against this null distribution. The significant ligand-receptor interactions identified were then visualized using dot plots and heatmaps. This circular plot illustrates the interactions between different cell types. The connections between cell types are represented by colored lines, indicating potential communication pathways or molecular interactions between distinct populations. These outputs were used to nominate candidate communication axes and were not treated as causal evidence.

**Trajectory and lineage inference analyses**

RNA velocity was calculated in renal grafts using the RNA velocity methodology(4). This technique estimates the rate of transcriptional changes within each cell by analyzing the ratio of spliced to unspliced reads, employing default parameters. The loom file generated from the previous step served as the input. Cells that lacked sufficient UMIs or unspliced reads after the new mapping were excluded from the analysis. Visualization of the RNA velocity was performed using t-SNE embedding, and state-transition directionality was estimated through a Markov process. The analysis steps largely followed the protocols provided in the open-source repository (https://github.com/rajewsky-lab/planarian_lineage). RNA velocity was interpreted as an inferred transcriptional relationship rather than lineage tracing.

Trajectory analysis was performed using Monocle 2(5) to explore the dynamic differentiation pathways of cell populations. The pre-processed scRNA-seq data, including normalized gene expression matrices and cell cluster annotations, were used as input for Monocle 2. Highly variable genes were selected to define the trajectory, focusing on genes associated with differentiation and functional states. Cells were ordered along pseudotime by constructing a minimum spanning tree (MST) on the reduced-dimensional space generated using the DDRTree algorithm. Root and branch points were manually defined based on biological knowledge and known marker gene expression patterns. Differentially expressed genes along the trajectory were identified using Monocle's built-in functions, and clusters were assigned based on their expression dynamics. To visualize the results, cells were colored by pseudotime or cluster identity, and branch-specific trajectories were highlighted. Key marker genes were overlaid on the trajectory to support interpretation of state-associated trajectories. All analyses were performed using Monocle 2 in the R statistical environment, following standard workflows. Pseudotime ordering was not interpreted as direct proof of lineage conversion.

Partition-based graph abstraction (PAGA)(6) analysis was performed to investigate the connectivity and differentiation trajectories among cell populations. Pre-processed scRNA-seq data, including normalized gene expression matrices and cell cluster annotations, were used as input for PAGA analysis using the *Scanpy* package in Python. Highly variable genes were selected to construct a neighborhood graph of cells using the k-nearest neighbor (k-NN) algorithm. PAGA was applied to the graph to infer connectivity between cell clusters, representing the likelihood of direct transitions based on shared gene expression profiles. The PAGA(7) graph was visualized as an abstracted structure, with nodes representing clusters and edges denoting connectivity strength. To integrate pseudotime ordering, the PAGA-initialized trajectory was further refined by assigning a root node based on prior biological knowledge or marker gene expression. Pseudotime was computed along the inferred trajectory, and differential gene expression was analyzed along the pseudotime axis to identify key genes driving transitions between states.

**Cross-Species Single-Cell Integration with SATURN**

Human and mouse single-cell or single-nucleus datasets were processed within species before cross-species integration. SATURN was used to embed cells in a shared macrogene space derived from protein-language-model embeddings, enabling comparison of conserved transcriptional programs beyond strict one-to-one ortholog matching. Cell identities were assigned using canonical markers and species-specific analyses. The integrated embedding was used to identify broad cross-species correspondences; endothelial, epithelial, T-cell and myeloid findings were subsequently evaluated within species and across orthogonal modalities to reduce overinterpretation of the shared space.

SATURN outputs were interpreted as atlas-level evidence of conserved programs and not as quantitative equivalence between species or clinical subgroups.

**Patients and Tissue Samples**

The in-house human component included eight recipients with clinically diagnosed chronic allograft dysfunction (CAD) and four recipients with stable graft function. Four CAD graft explants obtained at transplant nephrectomy were used for single-nucleus RNA sequencing. Four additional CAD biopsies and four stable biopsies were used for Visium HD spatial transcriptomics and adjacent-section MALDI-MSI. All participants provided written informed consent, and the protocol was approved by the Institutional Review Board of the Second Affiliated Hospital of Nanjing Medical University (2023-SRFA-007). Clinical characteristics are summarized in Supplementary Tables S1 and S2.

**Supplementary Table S1. Clinical characteristics of the CAD graft-explant cohort used for single-nucleus RNA sequencing.**

The graft-explant cohort comprised four CAD recipients. Age, sex, time after transplantation, donor source, maintenance immunosuppression, serum creatinine and estimated glomerular filtration rate are summarized below.

| **Clinical variable** | **CAD cohort** | **Stable cohort** | **P value** |
| --- | --- | --- | --- |
| **Case number (n)** | 2 | 2 | NS |
| **Age (years, mean ± SD)** | 41.0 ± 4.0 | 37.5 ± 3.5 | NS |
| **Gender (Male / Female)** | 1/1 | 2/0 | NS |
| **BMI (kg/m2, mean ± SD)** | 21.8 ± 3.9 | 23.3 ± 2.5 | NS |
| Time after transplantation (years) | 10.5，13.5 | 7.3, 6.2 |  |
| **Primary/secondary transplant** | 2/0 | 2/0 | NS |
| **PRA before renal transplant (%)** | 0 | 0 | NS |
| **Donor source** |  |  | NS |
| **Living-related** | 2 | 2 |  |
| **Cadaveric** | 0 | 0 |  |
| **Immunosuppressive regimen** |  |  | NS |
| **Prednisone + MMF + Tac** | 2 | 2 |  |
| **Prednisone + MMF + CsA** | 0 | 0 |  |
| **Biochemical parameters** |  |  |  |
| **Serum creatinine**  **(μmol/L, mean ± SD)** | 480 ± 30 | 95 ± 10 |  |
| eGFR (mL/min/1.73 m², mean ± SD) | 18.5 ± 2.5 | 77.0 ± 7.1 |  |

**Supplementary Table S2. Clinical characteristics of CAD and stable biopsy cohorts used for spatial transcriptomics and spatial metabolomics.**

| **Clinical variable** | **CAD cohort** | **Stable cohort** | **P value** |
| --- | --- | --- | --- |
| **Case number (n)** | 4 | 4 | NS |
| **Age (years, mean ± SD)** | 36.0 ± 3.5 | 38.0 ± 5.0 | NS |
| **Gender (Male / Female)** | 4/0 | 3/1 | NS |
| **BMI (kg/m2, mean ± SD)** | 22.5 ± 4.5 | 23.5 ± 4.0 | NS |
| Time after transplantation (years, range) | 8.5 (6.0-12) | 3.5 (2.5-4.0) | <0.001 |
| **Primary/secondary transplant** | 4/0 | 4/0 | NS |
| **PRA before renal transplant (%)** | 0 | 0 | NS |
| **Donor source** |  |  | NS |
| **Living-related** | 4 | 4 |  |
| **Cadaveric** | 0 | 0 |  |
| **Immunosuppressive regimen** |  |  | NS |
| **Prednisone + MMF + Tac** | 4 | 4 |  |
| **Prednisone + MMF + CsA** | 0 | 0 |  |
| **Biochemical parameters** |  |  |  |
| **Serum creatinine**  **(μmol/L, mean ± SD)** | 410 ± 25 | 95 ± 12 | <0.001 |
| eGFR (mL/min/1.73 m², mean ± SD) | 25.0 ± 3.0 | 75.0 ± 5.5 | <0.001 |

Abbreviations: CAD, chronic allograft dysfunction; BMI, body mass index; PRA, panel-reactive antibody; MMF, mycophenolate mofetil; CsA, cyclosporine A; Tac, tacrolimus; eGFR, estimated glomerular filtration rate; SD, standard deviation; NS, not significant.

**Tissue Immunofluorescence and Multiplex Fluorescent Immunohistochemistry**

Fluorescent multiplex immunohistochemistry was performed on 4-μm formalin-fixed paraffin-embedded sections using an OPAL multiplex system (PerkinElmer) according to the manufacturer’s instructions. After deparaffinization, antigen retrieval and blocking, sections were incubated with primary antibodies overnight at 4°C, followed by the appropriate fluorophore or OPAL detection steps. For Figure S10, Fibronectin, LDHA and SLC22A7 were examined together with DAPI. Primary antibodies were obtained from Proteintech and used at the manufacturer-recommended working dilutions. Images were acquired under identical settings within each experiment and quantified using predefined regions of interest.

For conventional immunofluorescence, sections were incubated with primary antibodies overnight at 4°C and fluorophore-conjugated secondary antibodies for 1 h at room temperature. Nuclei were counterstained with DAPI, and images were acquired using an Olympus fluorescence microscope or Zeiss LSM 880 NLO confocal microscope.

**HK-2 Cell Culture and Pharmacological Treatment**

HK-2 human proximal tubular epithelial cells were obtained from ATCC and maintained in RPMI-1640 medium supplemented with fetal bovine serum (VivaCell) under standard humidified culture conditions at 37°C and 5% CO₂. For the validation experiment, cells were assigned to four treatment groups for 48 h. The groups received vehicle, recombinant human TGF-β1 (Sino Biological, 80116-RNAH; 5 ng/mL), 2-deoxy-D-glucose (2-DG; MedChemExpress, HY-13966; 1 mM), or TGF-β1 plus 2-DG. Experiments were performed independently three times.

**Cell Immunofluorescence**

Cells were fixed, permeabilized and blocked using standard procedures, then incubated overnight at 4°C with antibodies against E-cadherin or α-SMA (Proteintech) at the manufacturer-recommended working dilutions. After incubation with species-appropriate fluorophore-conjugated secondary antibodies, nuclei were counterstained with DAPI. Images were acquired using identical exposure settings, and relative fluorescence was quantified from predefined fields using ImageJ.

**Western Blotting and Densitometry**

Renal graft proteins were extracted in RIPA buffer containing protease inhibitors. Equal amounts of protein were separated by SDS-PAGE, transferred to PVDF membranes and incubated with Proteintech antibodies against Fibronectin, LDHA, α-SMA and β-actin at the manufacturer-recommended working dilutions. HRP-conjugated secondary antibodies and enhanced chemiluminescence were used for detection. Band intensity was quantified with ImageJ, normalized to β-actin and expressed relative to the Syn mean.

**Statistics for the Focused Validation Experiments**

Data are presented as mean ± SEM from three independent experiments or biological samples. The four-group HK-2 experiment was analyzed separately for each marker using one-way ANOVA followed by Tukey’s multiple-comparison test. Syn and Allo-CAD tissue measurements were compared using a two-tailed unpaired t-test. P < 0.05 was considered statistically significant.

**Scope of Inference**

RNA velocity, pseudotime, PAGA, CellChat/CellPhoneDB, GSVA, AUCell and scMetabolism were used to infer state relationships, predicted communication or relative pathway activity. These analyses do not independently establish lineage conversion, metabolic causality or functional ligand-receptor dependence. The HK-2/2-DG experiment provides focused pharmacological support for the tubular glycolysis axis but does not establish PT(S3)-specific in vivo causality.

**Reference**

1.	Feng D, Gui Z, Xu Z, Zhang J, Ni B, Wang Z, et al. Rictor/mTORC2 signalling contributes to renal vascular endothelial-to-mesenchymal transition and renal allograft interstitial fibrosis by regulating BNIP3-mediated mitophagy. Clin Transl Med. 2024;14(5):e1686.

2.	Jin S, Plikus MV, Nie Q. CellChat for systematic analysis of cell-cell communication from single-cell transcriptomics. Nat Protoc. 2025;20(1):180–219.

3.	Efremova M, Vento-Tormo M, Teichmann SA, Vento-Tormo R. CellPhoneDB: inferring cell-cell communication from combined expression of multi-subunit ligand-receptor complexes. Nat Protoc. 2020;15(4):1484–506.

4.	La Manno G, Soldatov R, Zeisel A, Braun E, Hochgerner H, Petukhov V, et al. RNA velocity of single cells. Nature. 2018;560(7719):494–8.

5.	Qiu X, Mao Q, Tang Y, Wang L, Chawla R, Pliner HA, et al. Reversed graph embedding resolves complex single-cell trajectories. Nat Methods. 2017;14(10):979–82.

6.	Wolf FA, Hamey FK, Plass M, Solana J, Dahlin JS, Göttgens B, et al. PAGA: graph abstraction reconciles clustering with trajectory inference through a topology preserving map of single cells. Genome Biol. 2019;20(1):59.

7.	Rosen Y, Brbić M, Roohani Y, Swanson K, Li Z, Leskovec J. Toward universal cell embeddings: integrating single-cell RNA-seq datasets across species with SATURN. Nat Methods. 2024;21(8):1492–500.
